# Molecular genetic basis of the primary emotions in young adults: an exploratory analysis of genetic polymorphisms across dopamine, serotonin, oxytocin, endogenous opioid, and neurotrophic factor pathways

**DOI:** 10.3389/fphar.2025.1675538

**Published:** 2025-11-12

**Authors:** Timotej Glavač, Maruša Barbo, Metka Ravnik-Glavač, Maja Zupančič, Vita Dolžan

**Affiliations:** 1 Department of Psychology, Faculty of Arts, University of Ljubljana, Ljubljana, Slovenia; 2 Pharmacogenetics Laboratory, Institute of Biochemistry and Molecular Genetics, Faculty of Medicine, University of Ljubljana, Ljubljana, Slovenia

**Keywords:** primary emotions, molecular genetics, dopamine, serotonin, oxytocin, brain-derived neurotrophic factor, endogenous opioids

## Abstract

**Introduction:**

Advances in affective neuroscience have unraveled the neurobiological underpinnings of primary emotions, making them suitable candidates for molecular genetic research. The aim of this study was to perform an exploratory molecular genetic association analysis of primary emotions in humans.

**Methods:**

A total of 333 young adults (*M*_*age*_ = 21.96 years, *SD* = 2.48; 56.8% female) participated in this study. Participants were recruited predominantly from a local university using a community sampling procedure. Data were collected *via* an online questionnaire (1ka.si) which primarily included a validated measure of the primary emotions, specifically the (Affective Neuroscience Personality Scales – Brief) and demographic information. Participants provided informed consent prior to completing the survey, and responses were anonymized. Following the survey, participants provided buccal swabs and their DNA was genotyped for 14 single nucleotide polymorphisms across five genes relevant to KEGG pathways, including dopamine (*COMT* rs4680, rs165815), serotonin (*TPH2* rs1843809, rs4290270, rs7305115, rs4570625), oxytocin (*OXTR* rs53576, rs968389, rs2268498), endogenous opioid (*OPRM1* rs1799971, rs677830), and neurotrophic factor (*BDNF* rs6265, rs28722151, rs11030101).

**Results:**

Our findings revealed several significant and nominally significant associations between genetic polymorphisms and primary emotions which showed a clear sex-specific pattern. In males, associations were found with the *COMT* and *TPH2* polymorphisms. Specifically, *COMT* rs4680 was associated with ANGER and SADNESS, *TPH2* rs1843809 with PLAY, rs7305115 with CARE, and rs4570625 with CARE and SADNESS. In females, the three *BDNF* polymorphisms were differentially associated with FEAR, SADNESS (rs28722151 and rs11030101), and ANGER (rs6265). In the total sample, interaction effects were also found between the two *OPRM1* polymorphisms (rs1799971 and rs677830) with SADNESS and SEEKING.

**Discussion:**

Overall, the present study identified several novel candidate genes which might be related to primary emotions in a sample of young adults. Although our findings should be considered preliminary, they may have important implications for personality research as well as clinical practice.

## Introduction

Emotional dysregulation and the accompanied mental distress are considered key features of affective disorders (Campbell-Sills and Barlow, 2007; Joormann and Siemer, 2014) and several psychiatric syndromes (Panksepp, 1998; Panksepp, 2006; D’Agostino et al., 2018; Kraiss et al., 2020; Paulus et al., 2021). Affective disorders encompass a range of mental health conditions characterized by disturbances in mood regulation and entail persistent and significant changes in mood states, impacting individual’s emotional wellbeing and functioning (Andlin-Sobocki and Wittchen, 2005; APA, 2013; Harrison et al., 2021; Janiri et al., 2021). Attaining a thorough comprehension of the neurobiological and genetic substrates underlying fundamental emotional processes is therefore paramount for better understanding and ameliorating mental distress. Affective Neuroscience Theory (ANT) (Panksepp, 1998) provides a promising conceptual framework for exploring these mechanisms. Research within the field of affective neuroscience has established evidence for seven primary emotional systems in mammalian brains (Panksepp, 1998; Panksepp, 2005).

Each of these emotional systems has been shaped through evolution and serves as an inherited tool for survival and enhancing fitness (Montag and Panksepp, 2017; Davis and Panksepp, 2018). These systems were identified through rigorous research on animal models, employing techniques such as electrochemical stimulation, neuropharmacological manipulation, and lesion induction, followed by the observation of the distinct behaviors that accompany the activation of each discrete system (Panksepp and Biven, 2012). Such experimental studies have allowed the precise identification of specific neuromodulators (substances that, together with neurotransmitters, regulate the inhibitory and excitatory responses of brain receptors (Rosen et al., 1989; Sasaki et al., 2011)) involved in the regulation of each emotional system, e.g., dopamine in SEEKING, substance P in ANGER, corticotropin-releasing hormone in FEAR, oxytocin and endogenous opioids in SADNESS and CARE, and dopamine and cannabinoids in PLAY (Panksepp, 2005; Siviy and Panksepp, 2011). The firm evolutionary grounding of these systems as well as the identified neurochemical pathways allows for further examination through neurogenetic analyses.

The SEEKING system motivates animals to explore and forage for essential resources such as food, water, and shelter. In humans, its activation is linked to curiosity, enthusiasm, and general motivation (Davis and Montag, 2019). This system is highly conserved across species and is even observable in simple organisms such as *C. elegans*, which possess only about 1,000 somatic cells (Ward et al., 2009). The FEAR system facilitates learning about environmental threats and strategies for avoiding bodily harm or death. In humans, it is associated with anxiety, worry, and fear. The RAGE system supports self-defense and protection of resources and can be triggered when an animal’s movement is physically restrained. In humans, activation manifests as irritability, frustration, and anger. The PANIC system drives infants to seek closeness with caregivers, often expressed through separation distress, sadness, and vocalizations such as crying. Complementing this, the CARE system promotes parental nurturing, rudimentary empathy, affiliation, and love, supporting attachment between parents and offspring. Mammals also possess the PLAY system, which facilitates social interaction beyond the parental context, particularly in youth. Its activation is associated with joy and happiness and helps young mammals acquire species-specific social skills, develop behavioral flexibility as well as the capacities essential for survival in later life stages and prepare for integration into broader social environments (Panksepp, 1998).

Endophenotypes facilitate the examination of neuroanatomical, neurochemical circuitry, and genetic-behavioral pathways pertinent to understanding the mechanisms of mental distress (Gould and Gottesman, 2006; Walters and Owen, 2007; Savitz and Drevets, 2009; Iacono, 2018). Specifically, they represent quantifiable traits spanning neurophysiological, emotional, motivational, and cognitive tendencies that serve as indispensable indicators in the investigation of mental disorders (Gottesman and Gould, 2003; Gould and Gottesman, 2006). Their significance lies in their potential to offer a more direct and nuanced understanding of the underlying genetic and neural substrates implicated in psychiatric conditions (Gould and Gottesman, 2006; Panksepp, 2006). The emotional endophenotypes delineated in the ANT may signify the most ancient evolutionary components of personality, as they are rooted in the subcortical brain (Montag and Panksepp, 2017), and might thus be particularly useful in clinical research (Panksepp, 1998).

The primary emotional systems also overlap with Ekman’s “universal emotions” as both fall into the category of the “basic emotion” theories within emotion research (Tracy and Randles, 2011). This is because researchers within this field subscribe to the view that the basic emotions are universally shared, innate responses that are hardwired in the brain. In this way both views are aligned with an evolutionary approach. Relatedly, Darwin also proposed such a view of emotion (Darwin, 1872). Ekman and colleagues classified anger, disgust (there has been discussion on considering disgust as a primary emotion (Panksepp, 2007; Tolchinsky et al., 2024), fear, happiness, sadness and surprise as universal emotions (Ekman et al., 1969; Ekman and Friesen, 1971). These roughly coincide with the emotions identified within ANT. The differences in the categorized emotions arise mostly due to differences in the methodologies employed. While ANT research focused on carefully crafted animal studies, the basic emotion work stems from research on humans. These studies showed that people across different cultures recognize and interpret the same facial expression for universal emotions with high accuracy (Ekman et al., 1969; Ekman and Friesen, 1971), that differences in expression can be mapped to distinct physiological changes (Ekman et al., 1983) that micro expressions, reveal concealed emotions (Ekman and Friesen, 1974), and that blind athletes produce the same facial expressions as sighted athletes (Matsumoto and Willingham, 2009). There is considerable overlap between the two models of basic emotions, and several efforts have been made to integrate these theoretical perspectives (Montag and Panksepp, 2016). Due to ANT research delineating the neural circuity and neurochemistry involved in generating affect in animal models, however, this approach is more appropriate for molecular genetic research (Panksepp, 2006).

Both the primary emotions and the endophenotypes overlap with the RDoC (Research Domain Criteria) framework (Insel et al., 2010; Cuthbert and Insel, 2013; Cuthbert, 2020). The RDoC is a classification approach to mental disorders that aims to address some of the short-comings of previous systems such as the DSM (Diagnostic and Statistical Manual of Mental Disorders) and ICD (International Statistical Classification of Diseases and Related Health Problems), namely that it focuses on empirically examined characteristics of mental disorders and ones which integrate contemporary findings from genetics, systems neuroscience and behavioral science (Hyman, 2007; Sanislow et al., 2010), to enable translating basic research from animal or human studies into coherent models of pathology or mechanism-based treatments. The primary emotions in many ways satisfy these criteria, as ANT is grounded in rigorous research involving animal models in which the neural circuity, the behaviors following the stimulation of these circuits as well as the neurochemistry and consequent genetic factors involved in the generation of affect in mammalian and avian species have been delineated (Panksepp, 1998; Anderzhanova et al., 2017; Davis and Montag, 2019). However, it is important to note that it should not be assumed that endophenotypes show simpler genetic architecture than those phenotypes contributing to disease susceptibility (Gottesman and Gould, 2003). Indeed, genetic studies of behavioral endophenotypes have faced similar criticisms as disease phenotypes, including very small effect sizes of individual variants, low statistical power in early studies, and frequent replication failures (Duncan and Keller, 2011; Rietveld et al., 2014).

To investigate the expression of primary emotions in human subjects, the Affective Neuroscience Personality Scales (ANPS) (Davis et al., 2003) were developed, and subsequently revised (Pingault et al., 2012; Barrett et al., 2013; Montag et al., 2021). The scales measure the six primary emotions, SEEKING, FEAR, ANGER, CARE, SADNESS, and PLAY, while LUST was excluded due to concerns that social desirability might affect the validity of the scale. The ANPS and its later versions facilitated research concerning the role of these evolutionarily grounded emotional endophenotypes in psychiatric disorders and molecular genetic studies in humans. A recent review of studies employing the ANPS indeed suggested the relevance of emotional endophenotypes in a broad range of clinical disorders, including bipolar disorder, depressive disorder, attention deficit hyperactivity disorder, substance use disorders, and personality disorders such as borderline, narcissistic, antisocial, schizoid, and schizotypal disorder (Brienza et al., 2023). Along with these associations, the distinct neurochemistry identified in the evolutionary homologous areas of the mammalian brain provides the basis for molecular genetic research in humans. In fact, genetic linkage studies have been proposed since ANT was first outlined (Panksepp, 2006), with Montag et al. recently calling for studies providing biological validation (Montag and Davis, 2018; Montag et al., 2021).

Evidence for notable genetic effects on the primary emotions in humans comes from behavioral genetics (twin studies) that have suggested heritability estimates from 0.31 for ANGER to 0.69 for PLAY (Montag et al., 2016). Molecular genetic studies related to primary emotions have been scarce but have yielded important findings. For example, the authors found an association between ANGER and a genetic marker of the *DARP-32* gene (Reuter et al., 2009), an interaction between the oxytocin receptor gene (*OXTR*) and a serotonin transporter (*5-HTTLPR*) on FEAR and SADNESS (Montag et al., 2011), an interaction between genes in the dopaminergic pathway (dopamine transporter *(DAT1)* and Catechol-O-methyltransferase *(COMT)* genes) on SADNESS (Felten et al., 2011), an association between ANGER and a genetic variant of the orexin/hypocretin gene (Harro et al., 2019), an influence of *5-HTTLPR* on extreme response styles in the ANPS (Plieger et al., 2014), moderation effects of SADNESS levels in association with *BDNF* polymorphisms and executive functions during a priming task (Sanwald et al., 2020), and CARE mediating the association between empathy and an oxytocin receptor polymorphism (Liu et al., 2021).

Experiments conducted on animal models of ANT (Panksepp, 1998) have enabled the identification of the specific neurochemistry and neuroanatomy of each primary emotional system, which aids in the selection of candidate genes. In search for novel candidate genes and polymorphisms, reasonable candidates include generalized neuromodulators such as dopamine, serotonin, norepinephrine, oxytocin, endogenous opioids and brain-derived neurotrophic factors. These are also the targets of biological psychiatric interventions that modulate the degree of information processing across the entire neural network (Jaber et al., 1996; Taylor et al., 2006; Watt and Panksepp, 2009; Artigas, 2013; Björkholm and Monteggia, 2016).

The dopaminergic, serotonergic, oxytocinergic, endogenous opioid and brain derived neurotrophic factor systems are all involved in the activation of the primary emotions and have been frequently, associated with mental health outcomes and broader psychosocial functioning (Caspi et al., 2011; Olff et al., 2013; Lin et al., 2014; Gatt et al., 2015). Dopamine, synthesized by dopaminergic neurons in the brain, is a neurotransmitter central to processes such as motivation, motor activity, affect regulation, and reward (Wise and Rompre, 1989; Panksepp, 1998; Berridge, 2007). Serotonin, which regulates a wide range of physiological, cognitive, and behavioral functions, plays a particularly important role in emotional regulation as well as social and cognitive behavior (Canli and Lesch, 2007; Serretti et al., 2007; Celada et al., 2013). Oxytocin, a neuropeptide generated in the hypothalamus (Dubois-Dauphin et al., 1992; Caba et al., 1996; Baribeau and Anagnostou, 2015), is especially important for social processes, including attachment, bonding, and maternal caregiving (Richard et al., 1991; Buchheim et al., 2009). Endogenous opioids, including endorphins, enkephalins, and dynorphins, are neuropeptides that modulate pain, stress responses, and reward processing, and they play a central role in social bonding and affiliative behavior (Machin and Dunbar, 2011; Nummenmaa and Tuominen, 2018). Brain-derived neurotrophic factor (BDNF), a key neurotrophin involved in neuronal survival, synaptic plasticity, and neurogenesis, has been implicated in learning, memory, and the regulation of mood and emotional functioning, with alterations linked to depression and other psychiatric conditions (Castrén and Rantamäki, 2010; Autry and Monteggia, 2012).

The aim of the present study was to perform an exploratory molecular genetic association analysis of emotional endophenotypes in humans, specifically in relation to the primary emotions. Therefore we examined associations between the six primary emotions and 14 single nucleotide polymorphisms (SNPs) across five genes involved in pathways of key neurotransmitters and neuromodulators which are implicated in the neurochemistry of the primary emotions as outlined in ANT (Panksepp, 1998). These are dopamine (Catechol-O-methyltransferase - *COMT* rs4680, rs165815), serotonin (Tryptophan hydroxylase 2 - *TPH2* rs1843809, rs4290270, rs7305115, rs4570625), oxytocin (oxytocin receptor gene, *OXTR* - rs53576, rs968389, rs2268498), endogenous opioid (opioid receptor mu 1 - *OPRM1* rs1799971, rs677830), and neurotrophic factor (brain derived neurotrophic factor - *BDNF* rs6265, rs28722151, rs11030101) in a sample of young adults. While the primary emotions have been mapped onto specific neurochemistry in mammals, they have not yet been examined in relation to the majority of the polymorphisms included in this study. Because this study was exploratory in nature and identifying linkages between phenotypic traits and candidate genes is notably challenging (Van Gestel and Van Broeckhoven, 2003), we did not formulate specific hypotheses regarding the relationship between the primary emotions and the included gene variants. Sex differences have been observed in the expression of primary emotions in humans as well as in the neurobiology of affective disorders (Pahlavan et al., 2008; Pingault et al., 2012; Özkarar-Gradwohl et al., 2014; Pedersen et al., 2014). For example, rates of depression and anxiety are higher in women, which may be partially related to sex and reproductive hormones such as estradiol and estrogen (Altemus et al., 2014; Rubinow and Schmidt, 2019; Bangasser and Cuarenta, 2021). Consequently, we also aimed to examine sex-specific differences between the primary emotions and the included single nucleotide polymorphisms.

## Materials and methods

### Participants and procedure

Slovenian university students were invited to participate in the study through advertisements at five different faculties (science, technical, medical and humanities studies) of the University of Ljubljana. The only inclusion criterion for participation was age (i.e., from 18 up to 30 years). There were no exclusion criteria. A raffle of five 50€ gift card coupons was also included in the study to incentivize participation. Student recruitment and data collection took place between 10.2.2023 and 30.5.2023. The study was approved by the National Medical Ethics Commission under code 0120-445/2022/6. Participants provided informed consent within the online assessment application before they filled out an online battery of assessments as part of a larger project, including the Slovenian adaptation (Glavač and Zupančič, 2024) of the Affective Neuroscience Personality Scales–Brief (Barrett et al., 2013; Glavač and Zupančič, 2024). Next, each participant provided a buccal swab. To match the provided buccal swab with the completed questionnaire, the respondents were asked to write a six-digit code from the online questionnaire on their buccal swab. Seventeen buccal swabs could not be identified or matched with the provided code and were excluded from the analysis. The final analysis included data from 333 (M_age_ = 21.96 years, *SD* = 2.479; 56.8% female) young adults.

### Psychological assessment measures

The Affective Neuroscience Personality Scales—Brief (BANPS) (Barrett et al., 2013) is a self-report measure designed to assess the expression of the six primary emotions in humans. The BANPS consists of 33 items that measure SEEKING (six items), PLAY (six items), CARE (four items), FEAR (five items), ANGER (six items) and SADNESS (six items). Responses are indicated along a 5-point rating scale (1 – strongly disagree; 5 – strongly agree). Three of these scales refer to positive emotions of SEEKING (e.g., “My curiosity drives me to do things.”), PLAY (e.g., “People who know me would say I am a very fun-loving person.”), and CARE (e.g., “I am the kind of person that likes to touch and hug people.”). The other three scales of FEAR (e.g., “I sometimes cannot stop worrying about my problems.”), ANGER (e.g. “When someone makes me angry, I tend to remain fired up for a long time”) and SADNESS (e.g., “I often feel lonely.”) represent the negative emotions. The BANPS has shown good construct and convergent validity (associations with positive and negative affect, emotion regulation and self-esteem), and internal consistency of each scale (Barrett et al., 2013). Similar psychometric properties were documented using the Slovenian adaptation of the BANPS. In addition, the scales also converged with theoretically congruent basic personality traits and suggested invariant measurement across sex (Glavač and Zupančič, 2024). The internal reliability of the scales (Cronbach’s alphas) in the present study ranged from 0.70 (CARE) to 0.86 (SADNESS).

### DNA isolation and genotyping

DNA extraction was carried out in accordance with the manufacturer’s guidelines using the QIAamp DNA Mini Kit (Qiagen, Venlo, Netherlands). The DNA concentration obtained from buccal swabs was assessed using the NanoDrop ND-1000 (Thermo Fisher Scientific, Waltham, MA, USA), and its purity was evaluated by measuring the absorbance ratio at 260/280 nm. The DNA isolation and genotyping took place between 10.8.2023 and 5.10.2023. The buccal swab samples were stored in the Pharmacogenetics laboratory at the Institute for Biochemistry and molecular genetics in a refrigerator at conditions between 4 °C and 8 °C. After the isolation procedure the preserved DNA was stored at −20 °C until it was utilized. DNA concentration in each sample was determined spectrophotometrically and 40 ng of DNA was used in each genotyping reaction. The genotyping rate approached 100%. Genotyping was performed for 14 single-nucleotide polymorphisms (SNPs) in 5 genes in relevant KEGG pathways; dopamine (*COMT* rs4680, rs165815), serotonin (*TPH2* rs1843809, rs4290270, rs7305115, rs4570625), oxytocin (*OXTR* rs53576, rs968389, rs2268498), endogenous opioid (*OPRM1* rs1799971, rs677830), and brain derived neurotrophic factor (*BDNF* rs6265, rs28722151, rs11030101). The selection of polymorphisms was based on the available literature, the polymorphisms’ potential functionality, and a minimum minor allele frequency of 0.05. The included polymorphisms were selected to represent genes whose products are essential components of the major neurochemical systems mediating primary emotions according to ANT: dopamine (*COMT*) with SEEKING, serotonin (*TPH2*) with FEAR and SADNESS, oxytocin (*OXTR*) with SADNESS and CARE, endogenous opioids (*OPRM1*) with SADNESS and CARE, and brain derived neurotrophic factor (*BDNF*) with PLAY (Panksepp, 1998; Davis and Montag, 2019). All investigated polymorphisms were genotyped using competitive allele-specific PCR (KASP assays, LGC Biosearch Technologies, Hoddesdon, UK), following the manufacturer’s guidelines. Any samples in which a clear genotype could not be determined were excluded from further analysis (2 samples for rs6265, 4 for rs28722141, 4 for rs11030101, 2 for rs4680, 3 for rs677830, 4 for rs1799971, and 1 each for rs53576, rs968389, and rs4570625). The genotype frequencies exhibited no significant deviation from Hardy-Weinberg equilibrium (HWE) in all cases (*p* < 0.05) and are presented in Table S1 of the Supplementary Material.

### Statistical analysis

One-way ANOVAs were used to assess differences between genotypes. Dominant, additive, and recessive genetic models were used for analyses depending on the genotype frequencies. The agreement of genotype frequencies with Hardy-Weinberg equilibrium was examined by a chi-squared test. Internal consistency of the BANPS scales was estimated by Cronbach’s alpha coefficients. Two-way ANOVAs were used to examine interactions between polymorphisms. To examine the potential effects of two polymorphisms simultaneously, interaction models were analyzed that included both polymorphisms and their interaction term to calculate the effect size and *p*-value for polymorphism interactions. All statistical tests were two-sided. The analyses were conducted on the total sample, as well as on males and females separately. An ANCOVA with age as a covariate was also conducted on the total sample to control for potential confounding effects. Since this study was exploratory in nature and aimed to identify potential associations between genetic polymorphisms and primary emotions, we applied a less conservative Bonferroni correction based on the number of independent polymorphisms tested. This approach was chosen to avoid Type II errors (false negatives) while maintaining statistical power. We acknowledge that this correction increases the possibility of false positives, but it is consistent with recommendations for exploratory research (Gao et al., 2008; Peters, 2009; Maher et al., 2013). In the genotype-phenotype association analyses *p*-values less than or equal to 0.0036 (0.05/14) were deemed statistically significant, while *p*-values between 0.0036 and 0.050 were regarded as nominally significant. In relation to the polymorphism Interaction analyses *p*-values less than or equal to 0.0071 were considered statistically significant, while *p*-values between 0.0071 and 0.050 were regarded as nominally significant. Where the homogeneity of variances assumption was violated In the ANOVA cases Welch’s robust statistics were reported. All statistical analyses were carried out in IBM SPSS Statistics, version 25.0 (IBM Corp, 2017).

## Results

### Genetic variability and the expression of the primary emotions

**FIGURE 1:**
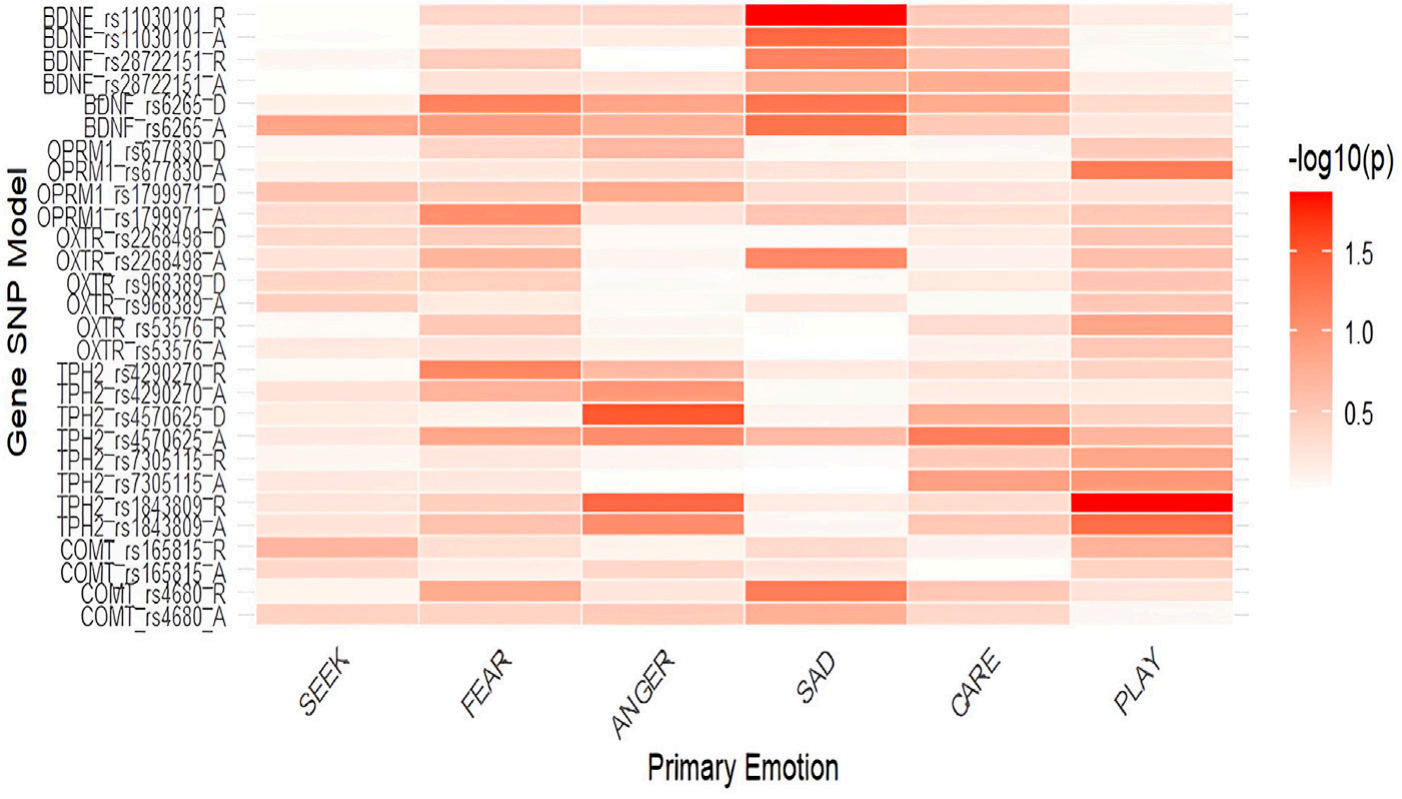
Heatmap displaying associations between the primary emotions and the included polymorphisms on the total sample (-log10(p)). *Notes*: SEEK–SEEKING, SAD–SADNESS. Heatmaps display the strength of association between SNPs and primary emotions, with color intensity representing the negative base-10 logarithm of *p*-values (−log_10_p); higher values indicate stronger statistical evidence for the association.

One-way ANOVAs were used to identify potential associations between polymorphisms and the six primary emotions. Associations between the included polymorphisms and the primary emotions are presented in Table 1 and Figure 1, while all mean scores are shown in Supplementary Table S2. Our main findings on the total sample show nominally lower levels of PLAY in homozygotes for the *TPH2* rs1843809 T allele in both the additive (*F* = 3.086; *p* = 0.047; η^2^ = 0.018) and recessive model (*F* = 6.166; *p* = 0.014; η^2^ = 0.018), as well as lower levels of ANGER in the recessive model (*F* = 4.088; *p* = 0.044; η^2^ = 0.012). Homozygotes for the *TPH2* rs4570625 G allele reported nominally higher levels of ANGER (*F* = 4.549; *p* =0 .034; η^2^ = 0.014) compared to GT heterozygotes and TT homozygotes. In addition, homozygotes for *BDNF* rs11030101 T allele reported nominally higher levels of SADNESS in the additive (*F* = 3.116; *p* = 0.046; η^2^ = 0.019) and recessive (*F* = 6.097; *p* = 0.014; η^2^ = 0.018) models. In the additive model of *OPRM1* rs1799971, calculations were not possible due to the small number of homozygotes for the polymorphic allele (Table 1). To examine for potential confounding effects, we also conducted the analyses on the total sample while controlling for age (Supplementary Table S5; Supplementary Figure S1). The analysis on the total sample showed that the results remained largely concordant even after controlling for age.

**TABLE 1:** Polymorphisms associated with the primary emotions on the total sample (N = 333).

Gene	SNP	Model	SEEK		FEAR		ANGER		SAD		CARE		PLAY	
			F	*P*	F	*P*	F	*P*	F	*P*	F	*p*	F	*p*
*COMT*	rs4680	A	0.938	0.393	0.931	0.395	1.100	0.334	2.046	0.179^a^	0.887	0.426^a^	0.159	0.853
		R	0.068	0.794	0.196	0.162	0.281	0.596	4.097	0.063^a^	1.049	0.306	0.319	0.573
	rs165815	A	0.803	0.449	0.345	0.709	0.851	0.428	0.497	0.609	0.038	0.963	0.945	0.390
		R	1.604	0.206	0.423	0.516	0.072	0.789	0.532	0.466	0.075	0.785	1.719	0.191
*TPH2*	rs1843809	A	0.561	0.571	1.309	0.272	2.429	0.090	0.153	0.858	1.182	0.308	3.086	**0.047**
		R	0.306	0.580	0.829	0.363	4.088	**0.044**	0.150	0.699	0.516	0.473	6.166	**0.014**
	rs7305115	A	0.444	0.642	0.469	0.626	0.034	0.966	0.006	0.994	2.028	0.133	2.221	0.110
		R	0.043	0.835	0.237	0.626	0.008	0.838	0.008	0.929	0.978	0.323	2.099	0.148
	rs4570625	A	0.452	0.637	1.927	0.147	2.392	0.093	1.431	0.240	2.343	0.066^a^	1.565	0.211
		D	0.194	0.660	0.077	0.782	4.549	**0.034**	0.065	0.799	1.687	0.183^a^	0.714	0.399
	rs4290270	A	0.604	0.547	1.627	0.198	2.209	0.111	0.097	0.908	0.366	0.694	0.428	0.652
		R	0.025	0.879^a^	3.127	0.078^a^	1.418	0.235	0.175	0.676	0.395	0.530	0.732	0.393
*OXTR*	rs53576	A	0.439	0.645	0.604	0.561^a^	0.211	0.810	0.006	0.994^a^	0.254	0.776	1.203	0.304^a^
		R	0.017	0.896	1.029	0.313^a^	0.030	0.862	0.005	0.942	0.479	0.489	2.184	0.142^a^
	rs968389	A	1.034	0.357	0.452	0.637	0.097	0.908	0.539	0.578	0.102	0.903	1.173	0.311
		D	0.662	0.416	0.824	0.365	0.007	0.935	0.018	0.892	0.200	0.655	1.088	0.298
	rs2268498	A	0.600	0.549	1.606	0.202	0.189	0.828	2.523	0.082	0.265	0.768	1.380	0.253
		D	0.617	0.433	0.908	0.341	0.021	0.886	0.009	0.926	0.175	0.676	1.164	0.281
*OPRM1*	rs1799971	D	0.572	0.450	2.889	0.090	0.356	0.551	1.067	0.302	0.419	0.518	1.031	0.311
		D	1.163	0.282	0.889	0.347	1.963	0.162	0.473	0.492	0.312	0.577	0.360	0.549
	rs677830	A	0.283	0.754	0.482	0.618	0.755	0.471	0.542	0.582	0.328	0.721	2.783	0.063
		D	0.049	0.825	0.651	0.420	1.497	0.222	0.035	0.853	0.042	0.848	0.963	0.327
*BDNF*	rs6265	A	1.985	0.139	2.163	0.117	1.691	0.186	2.945	0.054	1.157	0.316	0.495	0.610
		D	0.095	0.758	3.287	0.071	2.156	0.143	3.715	0.055	1.963	0.162	0.542	0.462
	rs28722151	A	0.046	0.955	0.603	0.548	0.553	0.576	1.692	0.186	1.795	0.168	0.341	0.711
		R	0.038	0.846	0.881	0.349	0.001	0.977	3.247	0.072	1.148	0.285	0.012	0.912
	rs11030101	A	0.078	0.925	0.340	0.712	0.379	0.685	3.116	**0.046**	1.172	0.311	0.151	0.860
		R	0.005	0.946	0.612	0.435	0.622	0.431	6.097	**0.014**	0.943	0.332	0.161	0.689

^a^ Robust Welch score reported if homogeneity of variances was violated. Significant (*p* < .0036) and nominally significant results (*p* = .0036–.05) are presented in bold. A-Additive model, R-Recessive model, D-Dominant model. Additive model was not calculated for *OPRM1* rs1799971 due to rare reference genotype. SEEK–SEEKING, SAD - SADNESS.

### Sex-specific differences

**FIGURE 2:**
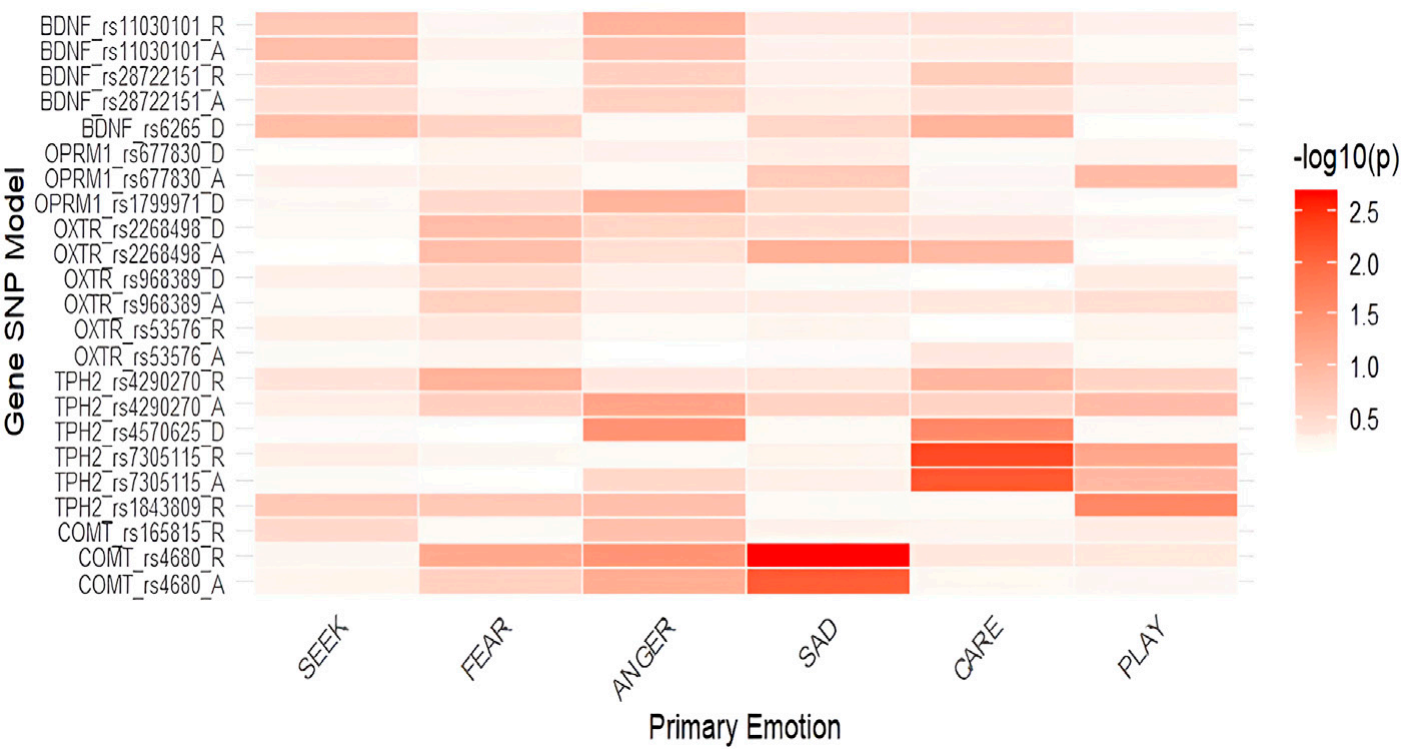
Heatmap of associations between primary emotions and genetic polymorphisms in males (-log10(p)). *Notes*: SEEK–SEEKING, SAD–SADNESS. Heatmaps display the strength of association between SNPs and primary emotions, with color intensity representing the negative base-10 logarithm of *p*-values (−log_10_p); higher values indicate stronger statistical evidence for the association.

**FIGURE 3:**
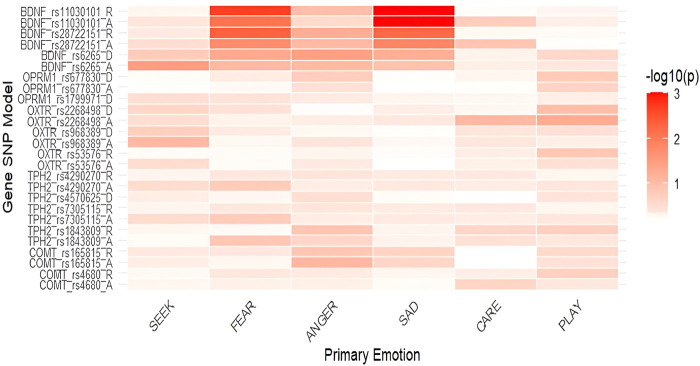
Heatmap of associations between primary emotions and genetic polymorphisms in females (-log10(p)). *Notes*: SEEK–SEEKING, SAD–SADNESS. Heatmaps display the strength of association between SNPs and primary emotions, with color intensity representing the negative base-10 logarithm of *p*-values (−log_10_p); higher values indicate stronger statistical evidence for the association.

Due to potential sex-specific effects, we also examined genetic associations in males and females separately. We observed sex-specific differences in the associations of several investigated polymorphisms (Table 2; Table 3 as well as Figure 2; Figure 3). All mean scores are presented in the Supplementary Material, Supplementary Table S3 (males) and Supplementary Table S4 (females). Several calculations in the additive model were not possible due to a low number of individuals with specific homozygous genotypes. In males, this was the case with *COMT* rs165815, *TPH2* rs4570625, *TPH2* rs1843809, *OPRM1* rs1799971, and *BDNF* rs6265 (Table 2) in females, these were *TPH2* rs4570625, and *OPRM1* rs1799971 (Table 3).

**TABLE 2:** Polymorphisms associated with the primary emotions in males (N = 142).

Gene	SNP	Model	SEEK		FEAR		ANGER		SAD		CARE		PLAY	
			F	*P*	F	*P*	F	*P*	F	*P*	F	*p*	F	*p*
*COMT*	rs4680	A	0.314	0.731	1.465	0.235	2.594	0.078	4.977	**0.008**	0.238	0.789	0.254	0.776
		R	0.093	0.761	2.657	0.067^a^	4.444	**0.037**	10.02	**0.002**	0.461	0.498	0.461	0.498
	rs165815	R	1.099	0.296	0.031	0.861	2.157	0.144	0.134	0.715	0.126	0.723	0.331	0.566
*TPH2*	rs1843809	R	1.852	0.176	1.742	0.189	2.196	0.141	0.023	0.879	0.054	0.816	5.157	**0.025**
	rs7305115	A	0.139	0.871	0.029	0.971	1.198	0.305	0.389	0.678	5.198	**0.007**	2.235	0.111
		R	0.270	0.604	0.059	0.808	0.032	0.859	0.140	0.709	8.020	**0.005**	3.501	0.063
	rs4570625	D	0.014	0.906	0.001	0.982	4.278	**0.035** ^a^	0.051	0.821	4.719	**0.027** ^a^	0.034	0.854
	rs4290270	A	0.495	0.611	1.452	0.238	2.894	0.059	1.366	0.259	1.353	0.262	2.108	0.125
		R	0.606	0.437	2.925	0.089	0.430	0.513	0.539	0.464	2.617	0.108	1.189	0.277
*OXTR*	rs53576	A	0.133	0.875	0.291	0.757^a^	0.030	0.971	0.114	0.892	0.689	0.504	0.190	0.827
		R	0.206	0.651	0.565	0.470^a^	0.039	0.844	0.104	0.748	0.002	0.964	0.141	0.708
	rs968389	A	0.198	0.820	1.289	0.238^a^	0.528	0.591	0.512	0.591^a^	0.705	0.496	0.968	0.375
		D	0.228	0.633	1.136	0.332^a^	0.244	0.622	0.030	0.872^a^	0.000	0.992	0.354	0.553
	rs2268498	A	0.037	0.963	2.240	0.128^a^	1.003	0.369	2.237	0.085^a^	2.177	0.117	0.052	0.949
		D	0.057	0.812	3.090	0.129^a^	1.242	0.267	0.975	0.374^a^	0.427	0.514	0.105	0.746
*OPRM1*	rs1799971	D	0.100	0.782^a^	1.029	0.312	2.869	0.093	0.922	0.338	0.072	0.789	0.000	0.985
	rs677830	A	0.369	0.692	0.458	0.633	0.152	0.859	1.655	0.195	0.224	0.799	2.143	0.121
		D	0.005	0.942	0.117	0.732	0.163	0.687	0.258	0.612	0.023	0.880	0.086	0.770
*BDNF*	rs6265	D	1.892	0.124^a^	1.156	0.268^a^	0.044	0.835	1.105	0.295	2.806	0.096	0.002	0.969
	rs28722151	A	1.049	0.353	0.287	0.751	1.470	0.234	0.501	0.607	0.880	0.417	0.327	0.722
		R	1.164	0.282	0.024	0.878	1.418	0.236	0.198	0.657	1.595	0.209	0.305	0.582
	rs11030101	A	2.061	0.131	0.379	0.685	1.993	0.140	0.395	0.675	0.562	0.571	0.177	0.838
		R	1.846	0.177	0.078	0.780	2.917	0.090	0.416	0.520	0.434	0.434	0.704	0.704

^a^ Robust Welch score reported if homogeneity of variances was violated. Significant (*p* < .0036) and nominally significant results (*p* = .0036–.05) are presented in in bold. A-Additive model, R-Recessive model, D-Dominant model. Several additive models for polymorphisms were not calculated due to rare homozygous genotypes of *COMT*, rs165815 CC, *TPH2* rs4570625 CC, *TPH2* rs1843809 GG, *OPRM1* rs1799971 GG, and *BDNF*, rs6265 AA. SEEK–SEEKING, SAD–SADNESS.

**TABLE 3:** Polymorphisms associated with the primary emotions in females (N = 191).

Gene	SNP	Model	SEEK		FEAR		ANGER		SAD		CARE		PLAY	
			F	*p*	F	*P*	F	*p*	F	*P*	F	*P*	F	*p*
*COMT*	rs4680	A	0.367	0.693	0.516	0.598	0.552	0.577	0.208	0.812	1.514	0.223	0.837	0.435
		R	0.089	0.766	0.640	0.425	0.503	0.479	0.165	0.685	0.341	0.560	1.662	0.199
	rs165815	A	0.635	0.531	0.353	0.703	2.567	0.080	1.409	0.247	0.044	0.957	1.041	0.355
		R	0.546	0.461	0.612	0.435	2.218	0.138	1.539	0.216	0.001	0.974	1.214	0.272
*TPH2*	rs1843809	A	0.220	0.803	2.009	0.137	1.419	0.245	0.440	0.645	1.014	0.365	0.842	0.432
		R	0.143	0.706	0.042	0.837	2.273	0.133	0.181	0.671	1.358	245	1.676	0.197
	rs7305115	A	1.238	0.292	1.809	0.167	0.632	0.533	0.743	0.477	0.630	0.534	0.857	0.426
		R	0.193	0.661	0.588	0.444	0.724	0.396	0.446	0.505	0.528	0.468	0.167	0.683
	rs4570625	D	0.395	0.530	0.121	0.728	0.998	0.319	0.035	0.852	0.001	0.977	0.710	0.400
	rs4290270	A	1.238	0.292	1.809	0.167	0.632	0.533	0.743	0.477	0.630	0.534	0.857	0.426
		R	0.193	0.661	0.588	0.444	0.724	0.396	0.446	0.505	0.528	0.468	0.167	0.683
*OXTR*	rs53576	A	1.219	0.298	0.192	0.825	0.739	0.479	0.021	0.979	0.565	0.569	0.110	0.336
		R	0.060	0.808	0.055	0.815	0.197	0.658	0.028	0.867	0.363	0.548	2.159	0.143
	rs968389	A	2.522	0.083	0.310	0.734	0.880	0.417	0.245	0.783	0.854	0.427	0.573	0.564^a^
		D	1.733	0.190	0.448	0.504	0.109	0.742	0.012	0.914	0.666	0.416	0.916	0.340
	rs2268498	A	1.199	0.304	0.444	0.642	0.597	0.551	0.778	0.461	2.474	0.087	2.958	0.054
		D	1.429	0.233	0.856	0.356	0.028	0.867	0.351	0.554	0.104	0.748	2.726	0.100
*OPRM1*	rs1799971	D	0.954	0.330	0.752	0.387	0.459	0.499	0.142	0.706	0.114	0.736	0.340	0.561
	rs677830	A	0.097	0.908	0.245	0.783	1.064	0.347	0.005	0.995	0.110	0.896	1.520	0.222
		D	0.033	0.857	0.446	0.505	1.879	0.172	0.010	0.922	0.135	0.714	2.019	0.157
*BDNF*	rs6265	A	3.491	**0.032**	2.459	0.088	2.495	0.085	2.117	0.123	0.601	0.549	0.859	0.425
		D	1.982	0.161	3.897	**0.050**	4.533	**0.035**	3.603	0.059	0.218	0.641	1.287	0.258
	rs28722151	A	1.154	0.318	3.201	**0.019** ^a^	2.445	0.090	4.276	**0**.**015**	1.984	0.140	0.088	0.916
		R	0.526	0.469	5.837	**0.005** ^a^	3.671	0.057	7.759	**0.006**	0.073	0.787	0.049	0.815
	rs11030101	A	0.875	0.418	3.968	**0.008** ^a^	1.325	0.268	8.552	**0.001**	1.721	0.182	0.535	0.586
		R	0.138	0.711	7.119	**0.002** ^a^	2.664	0.104	14.99	**0.001**	0.035	0.851	0.119	0.731

^a^ Robust Welch score reported if homogeneity of variances was violated. Nominally significant results (*p* = .0036–.05) are printed in gray, significant results (*p* <. 0.0036) are printed in bold. A-Additive model, R-Recessive model, D-Dominant model; Several additive models for polymorphisms were not calculated due to rare genotypes of *TPH2* rs4570625 CC, and *OPRM1* rs1799971 GG. SEEK–SEEKING, SAD–SADNESS.

In males (Table 2), nominally significant and significant differences were identified in *COMT* rs4680, specifically nominally in SADNESS in the additive (*F* = 4.977; *p* = 0.008; η^2^ = 0.012) and significantly in the recessive model (*F* = 10.018; *p* = 0.002; η^2^ = 0.067), with GG and GG + GA genotype carriers having higher scores than AA homozygotes. G allele carriers also reported nominally higher levels of ANGER (*F* = 4.444; *p* = 0.037; η^2^ = 0.031) in the recessive model. In the recessive model of *TPH2* rs1843809 nominally significant differences were found in PLAY, with GG + TG genotype carriers having higher scores than TT homozygotes (*F* = 5.157; *p* = 0.025; η^2^ = 0.035). Additionally, *TPH2* rs7305115 GG homozygotes had nominally lower levels of CARE in both the additive (*F* = 5.198; *p* = 0.007; η^2^ = 0.069) and recessive models (*F* = 8.020; *p* = 0.005; η^2^ = 0.054) compared to A allele carriers. In *TPH2* rs4570625, nominally significant differences were found in the dominant model for ANGER and CARE as GG homozygotes reported higher levels of ANGER (*F* = 4.278; *p* = 0.035; η^2^ = 0.030) and lower levels of CARE (*F* = 4.719; *p* = 0.027; η^2^ = 0.033).

In females (Table 3), differences were detected only for *BDNF* polymorphisms. In the dominant model, *BDNF* rs6265 GG homozygotes had nominally higher levels of FEAR (*F* = 3.897; *p* = 0.050; η^2^ = 0.021) and ANGER (*F* = 4.533; *p* = 0.035; η^2^ = 0.024) than A allele carriers. In the additive model *BDNF* rs28722151 heterozygotes (GC) had nominally lower levels of FEAR (*F* = 3.201; *p* = 0.019; η^2^ = 0.034) and SADNESS (*F* = 4.276; *p* = 0.015; η^2^ = 0.044), while in the recessive model GG homozygotes had nominally higher levels of FEAR (*F* = 5.837; *p* = 0.005; η^2^ = 0.031) and SADNESS (*F* = 7.759; *p* = 0.006; η^2^ = 0.040) compared to GC + CC genotypes. In *BDNF* rs11030101 TA heterozygotes had nominally lower levels of FEAR (*F* = 3.968; *p* =0 .008; η^2^ = 0.041) and significantly lower SADNESS (*F* = 8.552; *p* = 0.001; η^2^ = 0.085) in the additive model, while in the recessive model TT carriers had significantly higher levels of FEAR (*F* = 7.119; *p* = 0.002; η^2^ = 0.037) and SADNESS (*F* = 14.986; *p* = 0.001; η^2^ = 0.075).

### Effects of polymorphism-polymorphism interactions on the expression of the primary emotions

When examining the potential effects of interaction between polymorphisms, we found that carriers of *OPRM1* rs1799971 G allele (GA + GG), in combination with the *OPRM1* rs677830 T allele (TC + TT) reported significantly higher levels of SEEKING (*F* = 8.248; *p* = 0.004; ηp^2^ = 0.025) and nominally lower levels of SADNESS (*F* = 5.524; *p* = 0.019, ηp^2^ = 0.017) compared to the other three genotype combinations. Several interaction analyses were not possible due to the small sample size of some genotype groups (e.g. n < 10), as well as multicollinearity issues and were thus not reported (e.g., *TPH2* rs1843809 x rs7305115; rs6265 x rs11030101 and all three BDNF polymorphism interactions). Polymorphism interaction results are shown in Table 4.

**TABLE 4:** Interaction between polymorphisms in relation to the primary emotions.

Gene	Interaction	SEEK	FEAR	ANGER	SAD	CARE	PLAY
*COMT*	rs4680 x rs165815	0.059	0.922	0.341	0.852	0.183	0.615
*TPH2*	rs1843809 x rs4570625	0.438	0.304	0.530	0.258	0.380	0.251
	rs4290270 x rs7305115	0.700	0.680	0.967	0.810	0.297	0.750
	rs4290270 x rs4570625	0.845	0.995	0.870	0.425	0.824	0.204
*OXTR*	rs53576 x rs968389	0.568	0.835	0.826	0.119	0.389	0.409
	rs53576 x rs2268498	0.127	0.267	0.504	0.995	0.939	0.233
	rs968389 x rs2268498	0.123	0.867	0.475	0.142	0.301	0.209
*OPRM1*	rs1799971 x rs677830	**0.004**	0.218	0.094	**0.019**	0.311	0.794

*: Significant (*p* < .0071) and nominally significant results are presented in bold (*p* = .0071–0.05). SEEK–SEEKING, SAD–SADNESS.

## Discussion

The neurobiological underpinning of the primary emotions provides an opportunity to evaluate genetic risk and protective factors of various affective and psychiatric disorders. This study investigated common functional polymorphisms in candidate genes associated with relevant neuromodulators (dopamine, serotonin, endogenous opioids, oxytocin, and neurotrophic factors) that are related to primary emotions in a community sample of young adults. On the total sample our findings showed several significant and nominally significant differences in the expression of the primary emotions based on genetic variations in relevant pathways. These results remained largely consistent even after controlling for age, providing further robustness of the findings. Our study also revealed relevant sex-specific differences. We related our findings to previously reported polymorphisms associated with psychiatric disorders, specifically those linked to variations in primary emotions. In males, significant and nominally significant differences in the expression of primary emotions were found within *COMT* polymorphisms (ANGER and SADNESS in rs4680) and *TPH2* (PLAY in rs1843809, CARE in rs7305115 and ANGER and CARE in rs4570625), while in females, significant and nominally significant differences in the expression of primary emotions were identified in the three *BDNF* polymorphisms (FEAR and ANGER in rs6265, FEAR and SADNESS in rs28722151 as well as FEAR and SADNESS in rs11030101). We also identified interaction effects between two *OPRM1* polymorphisms in relation to SEEKING and SADNESS. Given the exploratory nature of this study, we discuss both significant and nominally significant results, with a focus on the more robust findings. Notably, both categories of results show effect sizes that suggest they warrant further consideration (Maher et al., 2013). However, as this study is exploratory, these findings should be viewed as indicative rather than confirmatory.

The activity of the COMT enzyme is primarily impacted by a functional rs4680 polymorphism, leading to a G to A substitution at codon 158 and results in a change from valine to methionine. The Val allele is associated with high COMT enzymatic activity, while the Met allele is associated with low activity, which results in higher dopamine levels (Lachman et al., 1996). Consequently, such individuals exhibit higher reward-seeking and report increased pleasure in response to positive events than individuals possessing a greater number of G alleles (Wichers et al., 2007; Lancaster et al., 2012). In this study we observed a robust sex-specific association with the rs4680 *COMT* polymorphism in SADNESS and a nominal association with ANGER both in the recessive model (lower levels in AA homozygotes compared to GG and CG). This effect was only observed in males. In relation to the primary emotions, elevated levels of depression have been associated with higher SADNESS, ANGER and FEAR (Montag et al., 2017). A large Swedish population based study on depressed individuals (Åberg et al., 2011) found a higher frequency of Met/Met and Met/Val genotypes in depressed individuals compared to controls, but only among men. In the only previous study assessing rs4680 with primary emotions (Felten et al., 2011), the authors found a significant association between the Met allele (A) and SADNESS and ANGER in a large sample of German university students, predominantly females. Several other studies have reported findings similar to ours, for example, an increased likelihood of early onset major depressive disorder among adults with the Val/Val genotype (Massat et al., 2005; Massat et al., 2011) as well as in depressive symptoms among children (Sheikh et al., 2013). Sex differences in *COMT* polymorphisms have commonly been reported (Tunbridge and Harrison, 2011) and have been attributed to the sexually dimorphic impact of *COMT* on estrogenic regulation Cohn and Axelrod (1971) as well as the modulation of COMT transcription by estrogen (Xie et al., 1999). However, it is unlikely that this is the sole mechanism accounting for gender-based dimorphism.

TPH serves as the rate-limiting enzyme in serotonin synthesis. The role of the serotonergic system in emotion regulation, including anger regulation and aggressiveness, has been well established (Waider et al., 2011; Lesch et al., 2012; Bortolato et al., 2013; Miczek et al., 2015), however in our study *TPH2* associations were only nominally significant. The *TPH2* rs4570625 polymorphism leads to a G to T base substitution in the promoter region at position −703, however the functionality of this polymorphism remains to be determined (Xiang et al., 2019). A study similar to ours, conducted on a sample of Estonian young adults found that rs4570625 TT homozygous males reported less aggressive behavior, lower levels of bullying perpetration, lower levels of maladaptive impulsivity and higher levels of adaptive impulsivity (Laas et al., 2017). These results are in line with ours as they also report higher levels of ANGER and lower levels of CARE in *TPH2* rs4570625 GG homozygotes in the dominant model. Higher levels of CARE have been linked to high agreeableness, a basic personality trait characterized by high levels of empathy, cooperative behavior and other prosocial features sustaining positive interpersonal relationships. However, TT carriers may be at greater risk for other disorders as a meta-analysis by Gao et al. (2012) identified T-allele carriers as having a heightened risk of affective disorders, while Mandelli et al. (2012) proposed that individuals with this genotype are more sensitive to stressful life events.

The *TPH2* rs1843809 polymorphism is a G > T intronic variant associated with attention deficit hyperactivity disorder (ADHD). Studies examining the expression of primary emotions in relation to ADHD indicate that the most common emotional endophenotype is characterized by lower levels of PLAY (Brienza et al., 2023). Accordingly, we found nominally lower levels of PLAY in *TPH2* rs1843809 in the recessive model (TT homozygotes), but only in males. Our results align with findings showing that individuals diagnosed with ADHD have a higher frequency of the T allele in Egyptian (Abo El Fotoh et al., 2020) and Korean children (Park et al., 2013) and in 179 Irish nuclear families (Sheehan et al., 2005). *TPH2* rs7305115 GG homozygotes also showed nominally lower levels of CARE in the recessive model. Social support is a key protective factor against suicidal risk (Kleiman and Liu, 2013) and individuals with lower levels of CARE may experience greater interpersonal difficulties, increasing their risk for ostracism and social exclusion (Hales et al., 2016) and the rs7305115 polymorphism has been implicated in suicide attempters (López-Narváez et al., 2015) and depression when combined with childhood abuse in G carriers (Li et al., 2023).

BDNF is highly expressed in the brain and is implicated in several neural processes, including neurogenesis, differentiation, survival, and synaptic plasticity. Stress and mood disorders are also known to decrease BDNF secretion (Mattson et al., 2004; Martinowich and Lu, 2008; Sen et al., 2008). The three investigated *BDNF* polymorphisms rs6265, rs28722151, and rs11030101 affect the levels of synthesized BDNF. These *BDNF* polymorphisms have been shown to be related to various mood disorders, including depression (Licinio et al., 2009). In the present study rs11030101 were robustly associated with primary emotions, while associations with rs6265 and rs28722151 were nominal.

The *BDNF* variant rs11030101 located at chr11:27659197 (GRCh38. p14) displays alleles A > G/A > T and is linked to an intron variant in the *BDNF* gene and a non-coding transcript variant in the *BDNF*-*AS* gene (Shkundin and Halaris, 2023). We found that TT homozygotes expressed higher levels of FEAR and SADNESS than AT heterozygotes and AA homozygotes in the recessive model, an effect that was found only in females. SADNESS, FEAR and ANGER are constitutive traits of neuroticism, one of the five basic personality traits (Montag and Davis, 2018), which is regarded as one of the major risk factors for depression and various psychopathology (Ormel et al., 2013; Barlow et al., 2021). Relatedly, BDNF is believed to play a key role in the mechanism of action of antidepressants due to its effects on neural plasticity (Castrén and Rantamäki, 2010). Previous molecular genetic studies have reported that rs11030101 is associated with negative symptoms in AA homozygote patients with schizophrenia (Ping et al., 2022), and higher levels of depression in A allele carriers (Licinio et al., 2009). In addition, higher AT heterozygote frequencies were identified in bipolar disorder and schizophrenia patients rather than in major depression patients and healthy controls (Pae et al., 2012).

The *BDNF* rs6265 polymorphism is characterized by the G > A substitution which results in the replacement of valine (Val) with methionine (Met) and includes a non-coding transcript variant within the BDNF-AS gene (Shkundin and Halaris, 2023). In our study, rs6265 A carriers had nominally lower levels of FEAR (*p* = 0.050) and ANGER (*p* = 0.035) in the dominant model (compared to heterozygotes and GG homozygotes), but this effect was only found in females. A study examining several *BDNF* polymorphisms, including rs6265 (Licinio et al., 2009), identified the G allele as a risk factor for development of the major depressive disorder (MDD). However, these findings are not conclusive, as several studies have reported A allele carriers to be at greater risk for depressive symptoms and neuroticism (Caldwell et al., 2013; Lehto et al., 2016; Aldoghachi et al., 2019). The last among the *BDNF* polymorphisms, i.e., rs28722151, leads to a *BDNF* intron variant and a non-coding transcript variant in the *BDNF-AS* gene (Urbina-Varela et al., 2020). We found that female *BDNF* rs28722151 GG homozygotes reported nominally higher levels of FEAR and SADNESS in the recessive model (compared to CG + CC). The only other reported study investigating this polymorphism in relation to psychopathology was conducted on a sample of Mexican Americans diagnosed with MDD and found that C allele carriers are at higher risk for developing MDD (Licinio et al., 2009).

Lastly, our findings also show interaction effects between the *OPRM1* rs1799971 and rs677830 polymorphisms. Rs1799971 is an A118G exon variant and rs677830 is a C to T substitution resulting in a premature stop codon at position 441. The importance of the endogenous opioid pathway in mental health has been implicated by Watt and Panksepp who proposed a theory of depression involving an endophenotype of low SEEKING and high SADNESS (Watt and Panksepp, 2009; Panksepp and Watt, 2011). The *OPRM1* gene plays a role in mitigating acute (short-term) psychosocial stress and negative mood in humans (Bershad et al., 2018). In primates *OPRM1* activation has been found to serve as a motivator for individuals to seek proximity with attachment figures when under stress, aiming to alleviate social pain (Barr et al., 2008). We found that carriers of the *OPRM1* rs1799971 GG genotype in combination with *OPRM1* rs677830 T allele reported significantly higher levels of SEEKING and lower levels of SADNESS. The rs1799971 AA/AG genotype has been associated with suicidal depression in outpatients diagnosed with depression (Nobile et al., 2021), treatment-emergent suicidal ideation (Nobile et al., 2019), and an increased risk of suicide (Hishimoto et al., 2008). The *OPRM1 rs*677830 polymorphism is novel in molecular genetic research and has been mostly examined in relation to opioid based medication and pain management response (Vidic et al., 2023). A recent study found that carriers of at least one rs677830 T allele experienced lower levels of neuropathic pain compared to AA homozygotes (Vidic et al., 2023). While neuropathic pain is not directly related to experiencing SADNESS, it has been associated with neuroticism and physical pain, as physical and psychological pain associated with SADNESS seems to share neural circuity (Macdonald and Leary, 2005).

### Characteristics and limitations of the study

The present study has several limitations. First, while our sample size is reasonably large within the context of candidate gene research in mental health, sample size remains a limitation. Power analysis conducted using G*Power (Faul et al., 2007) revealed that, with our sample size (n = 333), small to medium effects could be reliably identified. Regardless, a larger sample would increase statistical power, improving the likelihood of detecting true genetic associations while reducing the risk of false negatives. Relatedly, although several results were nominally significant, they did not withstand corrections, necessitating further replication studies between such polymorphisms and primary emotions. The issue of novel studies reporting considerably higher rates of significance compared to subsequent replication studies has been addressed as a notable challenge within molecular genetic research (Duncan and Keller, 2011). Subsequent replication studies are therefore needed to put our findings into perspective. Another limitation of the study is that we did not directly assess mental health phenotypes or psychopathological traits in our sample. Our interpretations are therefore based on prior literature linking the investigated SNPs to various mental disorders and personality traits. This approach is grounded in the view that primary emotions represent emotional endophenotypes (Montag and Davis, 2018) that are crucial for understanding vulnerability to mental health disorders (Gould and Gottesman, 2006).

A notable advantage of the study is its ethnically homogenous population, which provides valuable insights into the molecular genetic dynamics of primary emotions within this Central European context. This advantage stems from census data showing that Slovenia is relatively ethnically homogeneous, with most ethnic minority groups being from genetically and culturally similar neighboring populations (Surs, 2002). Ethnic homogeneity helps reduce confounding from population stratification in genetic analyses, which can potentially influence association results (Hellwege et al., 2017). Likewise, a shared cultural background reduces variability in the norms and practices of emotional expression, perception, and regulation as shown in previous cross-cultural studies (Özkarar-Gradwohl et al., 2020). Additionally, students from diverse fields of study were also included (science, technical, medical and humanities studies) to minimize the potential influence of personality traits (notably based on the primary emotional systems) on the results. While a degree of potential confounding effects were controlled for (the pattern of statistical significance on the total sample was largely consistent after controlling for age), we do not dismiss the possibility that other confounding factors may have also influenced the results. These may include the effects of mental disorders, medication use, or lifestyle variables such as smoking, substance use and physical activity (Sapranaviciute-Zabazlajeva et al., 2022; Johnstad, 2024). However, because our study involved a randomized community sample, the distribution of potential confounding variables such as mental disorders should reflect that of the general population within this age group (Gustavson et al., 2018) and as this study examines purely the genome level (SNPs), lifestyle variables are not expected to have as large an impact as they would in epigenetic research, where life experiences and behaviors play a more crucial role (Aristizabal et al., 2020). Further studies should also focus on other possibly relevant genetic pathways, such as those involved in sex hormones, as our study seems to point at pronounced sex-differences in the associations between the primary emotions and the SNPs. Polymorphisms in estradiol, testosterone and estrogen genes have been implicated in mental health disorders such as depression (Zarrouf et al., 2009; Hernández-Hernández et al., 2019). Furthermore, genes in the stress response system such as those related to cortisol were not included in this study but are very likely to influence the expression of the primary emotions and consequent disorders (Velders et al., 2011; Herbert, 2013).

## Conclusion

Overall, the present study was the first to examine several polymorphisms in dopamine, serotonin, oxytocin, endogenous opioid, and neurotrophic factor pathways in relation to the primary emotions using a relatively large-scaled general population of young adults with European ancestry. Our findings showed several nominally significant as well as significant associations between polymorphisms and primary emotions. These associations showed a clear sex-specific pattern. In males, associations were found in *COMT* and *TPH2* polymorphisms: *COMT* rs4680 was significantly associated with SADNESS (even after corrections) and nominally with ANGER, while *TPH2* rs1843809 was nominally linked to PLAY, rs7305115 to CARE, and rs4570625 to both CARE and SADNESS. In females, *BDNF* rs11030101 was strongly associated with FEAR and SADNESS, while *BDNF* rs28722151 and rs6265 showed nominal associations with FEAR, SADNESS, and ANGER, respectively. On the total sample, interaction effects were also found between the two *OPRM1* polymorphisms (rs1799971 and rs677830) in relation to SEEKING and SADNESS (nominally). Taken together, the present study can be considered an initial exploratory attempt in examining the potential influence of theoretically grounded molecular genetic pathways in relation to the primary emotions. However, future replication studies on larger samples are necessary to put these findings into context.

## Data Availability

The dataset employed in this study have been uploaded to the Open Science Framework depository and can be accessed at: https://osf.io/bnawp/overview?view_only=9290f173c93441cfae0a9c0032b008d3.
