# Supplementary material for: Molecular genetic basis of the primary emotions in young adults: an exploratory analysis of genetic polymorphisms across dopamine, serotonin, oxytocin, endogenous opioid, and neurotrophic factor pathways
Source: Front Pharmacol. 2025 Nov 12;16:1675538. doi: 10.3389/fphar.2025.1675538 (PMC12647084; doi:10.3389/fphar.2025.1675538)

**Supplementary Material**

**Table S1**. Hardy-Weinberg equation for all included polymorphisms

| Gene | SNP | Function Prediction | MAF | Alleles | | χ2 | *p* |
| --- | --- | --- | --- | --- | --- | --- | --- |
| COMT | rs4680 | Missense Variant | .49 | G | A | .311 | .856 |
| COMT | rs165815 | Missense Variant | .14 | C | T | 3.220 | .200 |
| TPH2 | rs1843809 | Intron Variant | .15 | G | T | .275 | .871 |
| TPH2 | rs7305115 | Synonymous Variant | .42 | A | G | .369 | .832 |
| TPH2 | rs4290270 | Synonymous Variant | .36 | A | T | 3.539 | .170 |
| TPH2 | rs4570625 | 2KB Upstream Variant | .20 | G | T | 2.606 | .272 |
| OXTR | rs53576 | Intron Variant | .33 | A | G | .241 | .887 |
| OXTR | rs968389 | Non Coding Variant | .33 | A | G | .260 | .878 |
| OXTR | rs2268498 | 2KB Upstream Variant | .44 | T | C | .242 | .886 |
| OPRM1 | rs1799971 | Missense Variant | .14 | A | G | 2.198 | .333 |
| OPRM1 | rs677830 | Stop Gained | .24 | C | T | .610 | .737 |
| BDNF | rs6265 | Missense Variant | .19 | G | A | .566 | .754 |
| BDNF | rs28722151 | Intron Variant | .47 | C | G | .312 | .856 |
| BDNF | rs11030101 | Non Coding Variant | .48 | A | T | .440 | .802 |

*Note*: The Hardy-Weinberg equilibrium analyses for each SNP were calculated by the Chi-square test. χ2 - Chi-square test. Significance values of *p* < .05 were considered consistent with the equilibrium.

**Table S2**. Primary emotion scores by genotype for total sample

| Gene | SNP | Genotype | | N | SEEK M (SD) | FEAR  M (SD) | ANGER M (SD) | SAD M (SD) | CARE M (SD) | PLAY M (SD) |
| --- | --- | --- | --- | --- | --- | --- | --- | --- | --- | --- |
| COMT | rs4680 | | GG | 76 | 18.38 **(**2.91) | 17.54 (4.38) | 16.64 (4.87) | 15.20 (4.14) | 14.34 (3.13) | 20.17 (3.18) |
|  |  | | GA | 170 | 18.96 (3.29) | 17.46 (4.57) | 15.65 (5.10) | 15.35 (4.63) | 14.71 (3.07) | 20.15 (3.24) |
|  |  | | AA | 85 | 18.88 (2.90) | 16.71 (4.74) | 15.64 (5.48) | 14.12 (5.26) | 15.00 (3.24) | 19.92 (3.29) |
|  |  | | GG + GA | 245 | 18.78 (3.19) | 17.51 (4.50) | 15.98 (5.04) | 15.31 (4.49) | 14.60 (3.09) | 20.16 (3.22) |
| COMT | rs165815 | | CC | 15 | 19.07 (3.63) | 16.47 (4.32) | 14.20 (4.84) | 15.47 (4.66) | 14.67 (3.98) | 19.40 (3.07) |
|  |  | | CT | 86 | 19.14 (3.00) | 17.13 (4.34) | 16.03 (5.00) | 14.57 (4.52) | 14.64 (3.25) | 19.78 (3.50) |
|  |  | | TT | 232 | 18.66 (3.12) | 17.38 (4.67) | 15.93 (5.20) | 15.11 (4.78) | 14.75 (3.04) | 20.23 (3.15) |
|  |  | | CC+CT | 101 | 19.13 (3.08) | 17.03 (4.33) | 15.76 (5.00) | 14.70 (4.53) | 14.64 (3.35) | 19.72 (3.42) |
| TPH2 | rs1843809 | | GG | 10 | 19.50 (2.59) | 15.80 (4.69) | 15.40 (5.72) | 15.70 (5.74) | 15.90 (1.55) | 20.90 (3.38) |
|  |  | | TG | 89 | 18.57 (3.28) | 17.83 (4.29) | 16.90 (5.55) | 15.08 (4.74) | 14.38 (3.50) | 20.73 (3.31) |
|  |  | | TT | 234 | 18.86 (3.07) | 17.13 (4.65) | 15.51 (4.91) | 14.92 (4.66) | 14.80 (3.02) | 19.79 (3.18) |
|  |  | | GG+TG | 100 | 18.66 (3.22) | 17.63 (4.35) | 16.75 (5.56) | 15.14 (4.83) | 14.53 (3.38) | 20.75 (3.30) |
| TPH2 | rs7305115 | | AA | 53 | 19.17 (2.89) | 16.91 (4.69) | 16.02 (5.63) | 15.04 (4.95) | 15.49 (3.13) | 20.85 (3.29) |
|  |  | | GA | 167 | 18.72 (2.96) | 27.50 (4.60) | 15.89 (5.08) | 14.99 (4.75) | 14.63 (2.91) | 20.07 (3.31) |
|  |  | | GG | 113 | 18.75 (3.42) | 17.11 (4.46) | 15.80 (5.01) | 14.96 (4.54) | 14.48 (3.40) | 19.72 (3.07) |
|  |  | | AA + GA | 220 | 18.83 (2.95) | 17.36 (4.62) | 15.92 (5.21) | 15.00 (4.79) | 14.84 (2.98) | 20.26 (3.32) |
| TPH2 | rs4290270 | | AA | 59 | 19.15 (2.96) | 17.44 (4.54) | 15.17 (4.96) | 15.00 (4.93) | 15.00 (2.75) | 20.32 (3.08) |
|  |  | | TA | 146 | 18.63 (2.89) | 17.70 (4.56) | 16.53 (5.42) | 15.10 (4.63) | 14.72 (3.32) | 20.14 (3.46) |
|  |  | | TT | 128 | 18.86 (3.41) | 16.72 (4.55) | 15.45 (4.82) | 15.85 (4.70) | 14.58 (3.08) | 19.88 (3.06) |
|  |  | | AA+TA | 205 | 18.78 (2.91) | 17.62 (4.54) | 16.14 (5.31) | 15.07 (4.71) | 14.80 (3.16) | 20.20 (3.35) |
| TPH2 | rs4570625 | | GG | 193 | 18.73 (3.27) | 17.23 (4.52) | 16.40 (5.17) | 14.95 (4.64) | 14.53 (3.34) | 20.22 (3.27) |
|  |  | | TG | 128 | 18.81 (2.94) | 17.59 (4.48) | 15.25 (5.03) | 15.28 (4.72) | 14.85 (2.80) | 19.79 (3.13) |
|  |  | | TT | 11 | 19.64 (2.06) | 14.82 (5.67) | 14.45 (5.16) | 12.82 (5.08) | 16.55 (2.62) | 21.36 (3.56) |
|  |  | | TG +TT | 139 | 18.88 (2.88) | 17.37 (4.62) | 15.19 (5.03) | 15.09 (4.78) | 15.00 (2.81) | 19.91 (3.18) |
| OXTR | rs53576 | | AA | 34 | 19.26 (2.86) | 16.74 (4.59) | 15.44 (5.47) | 14.94 (4.82) | 14.68 (2.99) | 20.09 (3.08) |
|  |  | | GA | 138 | 18.71 (3.06) | 17.11 (4.26) | 16.06 (5.30) | 15.01 (4.33) | 14.57 (2.98) | 20.38 (3.02) |
|  |  | | GG | 160 | 18.78 (3.22) | 17.54 (4.83) | 15.84 (4.95) | 14.96 (5.02) | 14.83 (3.30) | 19.80 (3.45) |
|  |  | | AA + GA | 172 | 18.82 (3.02) | 17.03 (4.31) | 15.94 (5.32) | 15.00 (4.41) | 14.59 (2.98) | 20.33 (3.03) |
| OXTR | rs968389 | | AA | 116 | 19.00 (2.92) | 17.57 (4.84) | 15.84 (4.86) | 14.93 (4.83) | 14.82 (3.11) | 19.82 (3.30) |
|  |  | | GA | 164 | 18.57 (3.24) | 17.04 (4.48) | 15.97 (5.30) | 14.82 (4.58) | 14.67 (3.26) | 20.35 (3.23) |
|  |  | | GG | 52 | 19.15 (3.10) | 17.25 (4.19) | 15.62 (5.33) | 15.60 (4.86) | 14.63 (2.80) | 19.77 (3.17) |
|  |  | | GA+GG | 216 | 18.71 (3.21) | 17.09 (4.40) | 15.88 (5.29) | 15.00 (4.65) | 14.66 (3.15) | 20.21 (3.21) |
| OXTR | rs2268498 | | TT | 106 | 18.99 (2.85) | 17.90 (4.86) | 16.12 (4.94) | 15.38 (4.93) | 14.88 (3.00) | 19.81 (3.28) |
|  |  | | TC | 161 | 18.61 (3.18) | 16.88 (4.55) | 15.80 (5.42) | 14.41 (4.55) | 14.68 (3.27) | 20.38 (3.28) |
|  |  | | CC | 66 | 18.97 (3.34) | 17.26 (4.00) | 15.68 (4.78) | 15.77 (4.58) | 14.53 (3.02) | 19.76 (3.05) |
|  |  | | TC + CC | 227 | 18.71 (3.23) | 16.99 (4.39) | 15.76 (5.23) | 14.78 (4.59) | 14.64 (3.19) | 20.20 (3.22) |
| OPRM1 | rs1799971 | | AA | 239 | 18.97 (3.00) | 17.41 (4.63) | 16.11 (5.18) | 15.08 (4.77) | 14.66 (3.11) | 20.16 (3.22) |
|  |  | | GA | 86 | 18.67 (3.20) | 16.95 (4.32) | 15.31 (5.07) | 14.79 (4.59) | 15.01 (3.15) | 20.01 (3.31) |
|  |  | | GA + GG | 90 | 18.55 (3.27) | 16.88 (4.39) | 15.22 (5.02) | 14.68 (4.59) | 14.88 (3.20) | 19.92 (3.34) |
| OPRM1 | rs677830 | | CC | 178 | 18.79 (3.18) | 17.47 (4.55) | 15.54 (5.04) | 14.95 (4.70) | 14.78 (3.10) | 19.94 (3.21) |
|  |  | | TC | 132 | 18.93 (3.02) | 17.00 (4.66) | 16.26 (5.36) | 14.89 (4.77) | 14.79 (3.15) | 20.51 (3.34) |
|  |  | | TT | 20 | 18.40 (2.62) | 17.60 (3.97) | 16.10 (4.18) | 16.05 (4.22) | 14.20 (3.22) | 18.85 (2.46) |
|  |  | | TC + TT | 152 | 18.86 (2.97) | 17.07 (4.57) | 16.24 (5.21) | 15.05 (4.70) | 14.71 (3.16) | 20.29 (3.28) |
| BDNF | rs6265 | | GG | 231 | 18.84 (3.12) | 17.58 (4.55) | 16.15 (5.00) | 15.31 (4.70) | 14.59 (3.15) | 20.00 (3.21) |
|  |  | | GA | 89 | 18.52 (3.03) | 16.75 (4.50) | 15.45 (5.37) | 14.47 (4.47) | 15.04 (3.07) | 20.21 (3.27) |
|  |  | | AA | 11 | 20.45 (2.46) | 15.27 (4.80) | 13.64 (5.85) | 12.27 (5.80) | 15.64 (2.50) | 20.91 (3.81) |
|  |  | | AG + AA | 100 | 18.73 (3.03) | 16.59 (4.53) | 15.25 (5.42) | 14.23 (4.65) | 15.11 (3.00) | 20.29 (3.32) |
| BDNF | rs28722151 | | CC | 82 | 18.85 (3.29) | 16.89 (4.15) | 16.39 (5.52) | 14.55 (4.55) | 14.38 (3.18) | 20.31 (3.09) |
|  |  | | GC | 159 | 18.75 (2.98) | 17.25 (4.77) | 15.65 (4.81) | 14.79 (4.72) | 15.04 (3.21) | 19.96 (3.42) |
|  |  | | GG | 88 | 18.86 (3.16) | 17.66 (4.60) | 15.89 (5.38) | 14.40 (2.85) | 14.40 (2.85) | 20.03 (3.05) |
|  |  | | GC + CC | 241 | 18.79 (3.08) | 17.12 (4.56) | 15.90 (5.07) | 14.71 (4.66) | 14.81 (3.21) | 20.08 (3.31) |
| BDNF | rs11030101 | | AA | 75 | 18.93 (3.21) | 17.04 (4.22) | 16.20 (5.44) | 14.75 (4.71) | 14.48 (3.21) | 20.00 (3.28) |
|  |  | | TA | 157 | 18.76 (3.08) | 17.21 (4.76) | 15.93 (4.96) | 14.49 (4.58) | 15.00 (3.25) | 20.17 (3.36) |
|  |  | | TT | 97 | 18.79 (3.02) | 17.59 (4.55) | 15.53 (5.27) | 15.97 (4.83( | 14.46 (2.87) | 19.96 (3.05) |
|  |  | | AA + TA | 232 | 18.82 (3.12) | 17.16 (4.58) | 16.02 (5.11) | 14.57 (4.61) | 14.84 (3.24) | 20.12 (3.33) |

*Note:* Raw primary emotion scale scores. M = Mean, SD = Standard deviation. SEEK – SEEKING, SAD – SADNESS.

**Table S3.** Primary emotion scores by genotype for males

| Gene | SNP | Genotype | n | SEEK M (SD) | FEAR  M (SD) | ANGER M (SD) | SAD M (SD) | CARE  M (SD) | PLAY M (SD) |
| --- | --- | --- | --- | --- | --- | --- | --- | --- | --- |
| COMT | rs4680 | GG | 29 | 19.21  (2.57) | 16.38  (4.65) | 15.24  (5.12) | 14.31  (4.31) | 13.86  (3.04) | 20.00  (3.62) |
|  |  | GA | 79 | 19.68  (3.28) | 15.87  (4.55) | 14.32  (5.09) | 14.39  (4.40) | 13.77  (3.09) | 20.15  (3.07) |
|  |  | AA | 34 | 19.74  (2.63) | 14.62  (3.54) | 12.53  (4.27) | 11.68  (4.25) | 14.21  (3.08) | 20.53  (2.88) |
|  |  | GG + GA | 108 | 19.56  (3.10) | 16.00  (4.56) | 14.56  (5.09) | 14.37  (4.35) | 13.80  (3.06) | 20.11  (3.21) |
| COMT | rs165815 | TT | 99 | 19.43  (2.94) | 15.71  (4.45) | 13.69  (4.84) | 13.64  (4.54) | 13.97  (2.91) | 20.33  (2.92) |
|  |  | CC+CT | 44 | 20.00  (3.07) | 15.57  (4.19) | 15.00  (5.14) | 13.93  (4.29) | 13.77  (3.39) | 19.98  (3.55) |
| TPH2 | s1843809 | TT | 100 | 19.83  (2.88) | 15.35  (4.35) | 13.69  (4.57) | 13.69  (4.33) | 13.87  (2.83) | 19.82  (3.13) |
|  |  | GG+TG | 43 | 19.09  (3.18) | 16.40  (4.33) | 15.02  (5.70) | 13.81  (4.77) | 14.00  (3.57) | 21.09  (2.93) |
| TPH2 | rs7305115 | AA | 19 | 19.58  (3.24) | 15.74  (3.80) | 12.47  (4.59) | 14.37  (4.21) | 15.32  (3.43) | 21.16  (2.85) |
|  |  | GA | 79 | 19.51  (2.75) | 15.72  (4.21) | 14.42  (4.96) | 13.46  (4.31) | 14.16  (2.79) | 20.38  (3.25) |
|  |  | GG | 45 | 19.80  (3.29) | 15.53  (4.89) | 14.20  (5.07) | 13.93  (4.84) | 12.87  (3.07) | 19.49  (2.90) |
|  |  | AA + GA | 98 | 19.52  (2.84) | 15.72  (4.11) | 14.04  (4.92) | 13.63  (4.28) | 14.39  (2.94) | 20.53  (3.18) |
| TPH2 | rs4290270 | AA | 26 | 19.77 (2.94) | 16.15 (3.81) | 12.42 (3.72) | 12.85 (4.13) | 14.38 (2.80) | 21.31 (2.56) |
|  |  | TA | 65 | 19.34  (2.82) | 16.12  (4.38) | 15.05  (5.39) | 14.37  (4.64) | 14.15  (3.07) | 20.06  (3.30) |
|  |  | TT | 52 | 19.87 (3.21) | 14.85 (4.54) | 13.73 (4.73) | 13.37 (4.64) | 13.37 (3.13) | 19.83 (3.07) |
|  |  | AA+TA | 91 | 19.46 (2.85) | 16.13 (4.20) | 14.30 (5.09) | 13.93 (4.35) | 14.22 (2.98) | 20.42 (3.14) |
| TPH2 | rs4570625 | GG | 82 | 19.61 (3.14) | 15.68 (4.66) | 14.84 (5.26) | 13.70 (4.78) | 13.45 (3.23) | 20.28 (3.12) |
|  |  | TG +TT | 60 | 19.55 (2.76) | 15.70 (3.95) | 13.12 (4.38) | 13.87 (3.97) | 14.57 (2.71) | 20.18 (3.10) |
| OXTR | rs53576 | AA | 15 | 19.33 (2.82) | 15.27 (3.41) | 14.33 (4.89) | 13.47 (3.85) | 14.73 (3.03) | 19.93 (2.96) |
|  |  | GA | 64 | 19.55 (2.83) | 15.45 (3.69) | 14.12 (5.47) | 13.92 (4.08) | 13.70 (2.97) | 20.38 (3.03) |
|  |  | GG | 64 | 19.73 (3.19) | 15.97 (5.13) | 14.00 (4.48) | 13.59 (4.96) | 13.92 (3.16) | 20.09 (3.27) |
|  |  | AA + GA | 79 | 19.51 (2.81) | 15.42 (3.62) | 14.16 (5.33) | 13.84 (4.02) | 13.90 (2.99) | 20.29 (3.00) |
| OXTR | rs968389 | AA | 53 | 19.45 (3.10) | 16.17 (5.27) | 14.36 (4.74) | 13.81 (5.17) | 13.91 (3.09) | 20.00 (3.24) |
|  |  | GA | 67 | 19.78 (3.00) | 15.04 (3.72) | 13.66 (5.13) | 13.40 (3.99) | 13.69 (3.12) | 20.57 (3.19) |
|  |  | GG | 23 | 19.48 (2.73) | 16.30 (3.61) | 14.74 (5.02) | 14.48 (3.98) | 14.57 (2.83) | 19.61 (2.57) |
|  |  | GA+GG | 90 | 19.70 (2.92) | 15.37 (3.72) | 13.93 (5.10) | 13.68 (3.99) | 13.91 (3.05) | 20.32 (3.06) |
| OXTR | rs2268498 | TT | 46 | 19.52 (3.01) | 16.59 (5.43) | 14.76 (4.87) | 14.26 (5.29) | 14.15 (3.00) | 20.33 (3.29) |
|  |  | TC | 69 | 19.62 (3.08) | 14.90 (3.73) | 13.49 (5.11) | 12.90 (3.90) | 13.41 (3.21) | 20.14 (3.20) |
|  |  | CC | 28 | 19.71 (2.76) | 16.04 (3.52) | 14.46 (4.69) | 14.79 (3.98) | 14.75 (2.59) | 20.14 (2.69) |
|  |  | TC + CC | 97 | 19.65 (2.98) | 15.23 (3.69) | 13.77 (4.99) | 13.47 (3.99) | 13.79 (3.09) | 20.14 (3.05) |
| OPRM1 | rs1799971 | AA | 107 | 19.65 (2.76) | 15.88 (4.55) | 14.50 (5.05) | 13.93 (4.50) | 13.87 (2.92) | 20.21 (3.09) |
|  |  | GA + GG | 36 | 19.47 (3.59) | 15.03 (3.67) | 12.89 (4.51) | 13.11 (4.29) | 14.03 (3.46) | 20.19 (3.26) |
| OPRM1 | rs677830 | CC | 75 | 19.60 (3.00) | 15.81 (4.03) | 13.91 (4.72) | 13.57 (4.35) | 13.91 (3.09) | 20.31 (2.98) |
|  |  | TC | 56 | 19.77 (2.87) | 15.36 (4.76) | 14.34 (5.40) | 13.55 (4.66) | 14.09 (2.92) | 20.48 (3.34) |
|  |  | TT | 10 | 18.90 (3.03) | 16.70 (4.62) | 13.70 (3.65) | 16.20 (3.26) | 13.40 (3.81) | 18.30 (2.50) |
|  |  | TC + TT | 66 | 19.64 (2.89) | 15.56 (4.73) | 14.24 (5.13) | 13.95 (4.55) | 13.98 (3.05) | 20.15 (3.31) |
| BDNF | rs6265 | GG | 101 | 19.37 (3.17) | 15.94 (4.46) | 14.17 (4.84) | 13.99 (4.52) | 13.67 (2.99) | 20.22 (3.29) |
|  |  | AG + AA | 41 | 20.12 (2.38) | 15.07 (4.10) | 13.98 (5.33) | 13.12 (4.31) | 14.61 (3.09) | 20.20 (3.40) |
| BDNF | rs28722151 | CC | 41 | 20.12 (2.91) | 15.29 (3.89) | 15.17 (5.29) | 13.10 (4.47) | 14.24 (3.01) | 20.51 (3.07) |
|  |  | GC | 55 | 19.53 (2.79) | 15.96 (4.45) | 13.91 (4.62) | 13.93 (4.44) | 13.98 (3.18) | 20.13 (3.29) |
|  |  | GG | 45 | 19.20 (3.27) | 15.56 (4.70) | 13.38 (5.06) | 13.93 (4.55) | 13.40 (2.91) | 19.98 (3.05) |
|  |  | GC + CC | 96 | 19.78 (2.84) | 15.68 (4.22) | 14.45 (4.93) | 13.57 (4.45) | 14.09 (3.10) | 20.29 (3.19) |
| BDNF | rs11030101 | AA | 34 | 20.50 (2.65) | 15.26 (4.00) | 15.29 (5.62) | 13.15 (4.49) | 14.35 (3.06) | 20.44 (3.21) |
|  |  | TA | 55 | 19.53 (3.01) | 16.05 (4.49) | 14.18 (4.51) | 13.75 (3.46) | 13.87 (3.29) | 20.13 (3.21) |
|  |  | TT | 52 | 19.19 (3.11) | 15.54 (4.52) | 13.13 (4.89) | 14.02 (4.48) | 13.63 (2.86) | 20.04 (3.03) |
|  |  | AA + TA | 89 | 19.90 (2.90) | 15.75 (4.30) | 14.61 (4.96) | 13.52 (4.45) | 14.06 (3.20) | 20.25 (3.20) |

*Note:* Raw primary emotion scores. M = Mean, SD = Standard deviation. SEEK – SEEKING, SAD – SADNESS.

**Table S4**. Mean and standard deviation for primary emotion scores per genotype configuration of females

| Gene | SNP | Genotype | n | SEEK M (SD) | FEAR  M (SD) | ANGER M (SD) | SAD M (SD) | CARE  M (SD) | PLAY M (SD) |
| --- | --- | --- | --- | --- | --- | --- | --- | --- | --- |
| COMT | rs4680 | GG | 47 | 17.87 (3.01) | 18.26 (4.08) | 17.51 (4.56) | 15.74 (3.99) | 14.64 (3.18) | 20.28 (2.92) |
|  |  | GA | 90 | 18.32 (3.21) | 18.81 (4.16) | 16.88 (4.82) | 16.19 (4.72) | 15.53 (2.85) | 20.20 (3.38) |
|  |  | AA | 51 | 18.31 (2.96) | 18.10 (4.94) | 17.71 (5.24) | 15.75 (5.27) | 15.53 (3.26) | 19.53 (3.47) |
|  |  | GG + GA | 136 | 18.16 (3.15) | 18.67 (4.10) | 17.14 (4.72) | 16.06 (4.48) | 15.23 (2.99) | 20.23 (3.23) |
| COMT | rs165815 | CC | 10 | 19.20 (3.61) | 17.70 (4.24) | 14.10 (5.09) | 16.80 (4.64) | 15.60 (3.31) | 18.70 (2.91) |
|  |  | CT | 46 | 18.28 (2.83) | 18.17 (4.14) | 16.96 (4.68) | 14.96 (4.70) | 15.28 (3.22) | 19.78 (3.42) |
|  |  | TT | 133 | 18.08 (3.14) | 18.63 (4.44) | 17.59 (4.84) | 16.21 (4.67) | 15.32 (3.01) | 20.17 (3.32) |
|  |  | CC+CT | 56 | 18.45 (2.97) | 18.09 (4.12) | 16.45 (4.84) | 15.28 (4.70) | 15.34 (3.21) | 19.59 (3.34) |
| TPH2 | rs1843809 | GG | 8 | 18.88 (2.53) | 15.75 (5.06) | 16.88 (5.44) | 14.88 (6.10) | 15.75 (1.67) | 20.63 (3.74) |
|  |  | TG | 48 | 18.23 (3.35) | 19.04 (3.84) | 18.27 (5.10) | 16.38 (4.41) | 14.79 (3.39) | 20.46 (3.57) |
|  |  | TT | 133 | 18.14 (3.03) | 18.43 (4.44) | 16.91 (4.71) | 15.84 (4.71) | 15.50 (2.99) | 19.80 (3.22) |
|  |  | GG+TG | 56 | 18.32 (3.24) | 18.57 (4.15) | 18.07 (5.12) | 16.16 (4.65) | 14.93 (3.21) | 20.48 (3.56) |
| TPH2 | rs7305115 | AA | 34 | 18.94 (2.71) | 17.56 (5.05) | 18,00 (5.22) | 15.41 (5.35) | 15.59 (3.00) | 20.68 (3.54) |
|  |  | GA | 87 | 18.00 (2.99) | 19.08 (4.38) | 17.28 (4.83) | 16.38 (4.76) | 15.06 (2.98) | 19.84 (3.36) |
|  |  | GG | 68 | 18.06 (3.54) | 18.14 (3.84) | 16.85 (4.72) | 15.63 (4.24) | 15.54 (3.21) | 19.87 (3.19) |
|  |  | AA + GA | 121 | 18.26 (2.93) | 18.65 (4.61) | 17.48 (4.93) | 16.11 (4.93) | 15.21 (2.98) | 20.07 (3.41) |
| TPH2 | rs4290270 | AA | 34 | 18.94 (2.71) | 17.56 (5.05) | 18.00 (5.22) | 15.41 (5.35) | 15.59 (3.00) | 20.68 (3.54) |
|  |  | TA | 87 | 18.00 (2.99) | 19.08 (4.38) | 17.28 (4.83) | 16.38 (4.76) | 15.06 (2.98) | 19.84 (3.56) |
|  |  | TT | 68 | 18.06 (3.35) | 18.15 (3.84) | 16.85 (4.72) | 15.63 (4.24) | 15.54 (3.21) | 19.87 (3.19) |
|  |  | AA+TA | 121 | 18.26 (2.93) | 18.65 (4.61) | 17.48 (4.93) | 16.11 (4.93) | 15.21 (2.98) | 20.07 (3.42) |
| TPH2 | rs4570625 | GG | 111 | 18.07 (3.22) | 18.38 (4.07) | 17.55 (4.80) | 15.88 (4.33) | 15.33 (3.20) | 20.17 (3.40) |
|  |  | TG +TT | 78 | 18.36 (2.90) | 18.60 (4.74) | 16.83 (4.92) | 17.01 (5.18) | 15.32 (2.88) | 19.76 (3.23) |
| OXTR | rs53576 | AA | 19 | 19.21 (2.97) | 17.89 (5.13) | 16.32 (5.87) | 16.11 (5.28) | 14.63 (3.04) | 20.21 (3.24) |
|  |  | GA | 74 | 17.99 (3.09) | 18.54 (4.21) | 17.73 (4.55) | 15.96 (4.34) | 15.32 (2.80) | 20.39 (3.03) |
|  |  | GG | 95 | 18.13 (3.11) | 18.56 (4.33) | 17.13 (4.87) | 15.87 (4.90) | 15.45 (3.28) | 19.64 (3.57) |
|  |  | AA + GA | 93 | 18.24 (3.09) | 18.41 (4.39) | 17.44 (4.85) | 15.90 (4.51) | 15.18 (2.85) | 20.35 (3.06) |
| OXTR | rs968389 | AA | 63 | 18.62 (2.72) | 18.75 (4.12) | 17.08 (4.64) | 15.87 (4.34) | 15.59 (2.94) | 19.67 (3.36) |
|  |  | GA | 96 | 17.72 (3.17) | 18.39 (4.47) | 17.64 (4.80) | 15.79 (4.75) | 15.35 (3.22) | 20.24 (3.25) |
|  |  | GG | 29 | 18.90 (3.41) | 18.00 (4.51) | 16.31 (5.55) | 16.48 (5.36) | 14.69 (2.83) | 19.90 (3.61) |
|  |  | GA+GG | 125 | 18.20 (3.09) | 18.30 (4.61) | 17.33 (4.99) | 15.95 (4.88) | 15.20 (3.13) | 20.16 (3.32) |
| OXTR | rs2268498 | TT | 60 | 18.58 (2.68) | 18.90 (4.15) | 17.17 (4.77) | 16.23 (4.48) | 15.43 (2.90) | 19.42 (3.25) |
|  |  | TC | 91 | 17.84 (3.08) | 18.32 (4.58) | 17.59 (4.98) | 15.50 (4.74) | 15.66 (3.00) | 20.60 (3.32) |
|  |  | CC | 38 | 18.42 (3.64) | 18.16 (4.14) | 16.58 (4.70) | 16.50 (4.91) | 14.37 (3.33) | 19.47 3.29) |
|  |  | TC + CC | 129 | 18.00 (3.25) | 18.27 (4.43) | 17.29 (4.91) | 15.79 (4.78) | 15.28 (3.14) | 20.27 (3.34) |
| OPRM1 | rs1799971 | AA | 132 | 18.41 (3.09) | 18.65 (4.32) | 17.42 (4.93) | 16.01 (4.81) | 15.30 (3.12) | 20.13 (3.33) |
|  |  | GA + GG | 53 | 17.92 (2.95) | 18.04 (4.43) | 16.89 (4.75) | 15.72 (4.56) | 15.47 (2.93) | 19.81 (3.40) |
| OPRM1 | rs677830 | CC | 102 | 18.19 (3.20) | 18.65 (4.56) | 16.79 (4.94) | 15.95 (4.74) | 15.43 (2.98) | 19.70 (3.35) |
|  |  | TC | 76 | 18.32 (3.00) | 18.18 (4.23) | 17.67 (4.91) | 15.88 (4.64) | 15.30 (3.23) | 20.53 (3.36) |
|  |  | TT | 10 | 17.90 (2.18) | 18.50 (3.17) | 18.50 (3.27) | 15.90 (5.20) | 15.00 (2.45) | 19.40 2.41) |
|  |  | TC + TT | 86 | 18.27 (2.91) | 18.22 (4.10) | 17.77 (4.74) | 15.88 (4.68) | 15.27 (3.14) | 20.40 (3.28) |
| BDNF | rs6265 | GG | 130 | 18.44 (3.03) | 18.85 (4.21) | 17.69 (4.57) | 16.34 (4.60) | 15.30 (3.10) | 19.84 (3.35) |
|  |  | GA | 49 | 17.37 (3.12) | 17.86 (4.57) | 16.53 (5.26) | 15.22 (4.69) | 15.31 (3.08) | 20.31 (3.20) |
|  |  | AA | 9 | 19.78 (2.05) | 16.00 (4.39) | 14.56 (5.88) | 13.67 (5.45) | 16.44 (1.88) | 21.11 (3.72) |
|  |  | AG + AA | 57 | 17.75 (3.12) | 17.49 (4.56) | 16.07 (5.27) | 14.93 (4.82) | 15.53 (2.95) | 20.44 (3.30) |
| BDNF | rs28722151 | CC | 41 | 17.59 (3.19) | 18.49 (3.80) | 17.61 (5.54) | 16,00 (4.19) | 14,51 (3.37) | 20.12 (3.15) |
|  |  | GC | 103 | 18.34 (3.02) | 17.88 (4.82) | 16.63 (4.67) | 15.24 (4.85) | 15.61 (3.10) | 19.90 (3.49) |
|  |  | GG | 43 | 18.51 (3.05) | 19.86 (3.32) | 18.51 (4.38) | 17.67 (4.25) | 15.44 (2.39) | 20.09 (3.08) |
|  |  | GC + CC | 144 | 18.13 (3.07) | 18.06 (4.55) | 16.91 (4.93) | 15.46 (4.67) | 15.30 (3.21) | 19.97 (3.39) |
| BDNF | rs11030101 | AA | 41 | 17.63 (3.08) | 18.51 (3.84) | 16.95 (5.24) | 16.07 (4.52) | 14.59 (3.37) | 19.63 (3.34) |
|  |  | TA | 101 | 18.35 (3.07) | 17.79 (4.81) | 16.93 (4.94) | 14.88 (4.63) | 15.62 (3.08) | 20.24 (3.44) |
|  |  | TT | 45 | 18.33 (2.89) | 19.96 (3.30) | 18.29 (4.28) | 18.22 (4.23) | 15.42 (2.59) | 19.87 (3.10) |
|  |  | AA + TA | 142 | 18.14 (3.08) | 18.00 (4.55) | 16.94 (5.01) | 15.23 (4.61) | 15.32 (3.19) | 20.06 (3.41) |

*Note:* Raw primary emotion scores. M = Mean, SD = Standard deviation. SEEK – SEEKING, SAD – SADNESS.

**Table S5**. Associations between the primary emotions on the total sample while controlling for age (*N* = 333)

| Gene | SNP | Model | SEEK | | | | | FEAR | | ANGER | | SAD | | CARE | | | PLAY | | | |
| --- | --- | --- | --- | --- | --- | --- | --- | --- | --- | --- | --- | --- | --- | --- | --- | --- | --- | --- | --- | --- |
|  |  |  | F | | *P* | | F | | *P* | F | *P* | F | *P* | | F | *p* | | F | *p* | |
| *COMT* | rs4680 | A | .734 | .481 | | .770 | | | .464 | 1.003 | .368 | 1.948 | .144 | | 1.007 | .366 | | .140 | | .869 |
|  |  | R | .477 | .490 | | 1.656 | | | .199 | .041 | .840 | 3.990 | **.047** | | 1.262 | .262 | | .279 | | .597 |
|  | rs165815 | A | .546 | .580 | | | .274 | | .760 | .669 | .513 | .696 | .499 | | .212 | .809 | | 1.226 | | .295 |
|  |  | R | .873 | .351 | | .390 | | | .533 | .355 | .552 | .441 | .507 | | .326 | .569 | | 2.160 | | .143 |
| *TPH2* | rs1843809 | A | 1.012 | .365 | | 1.131 | | | .324 | 2.996 | **.051** | .193 | .825 | | 1.007 | .367 | | 2.991 | | **.052** |
|  |  | R | .635 | .426 | | .430 | | | .512 | 4.101 | **.044** | .157 | .692 | | .254 | .614 | | 6.184 | | **.013** |
|  | rs7305115 | A | .438 | .646 | | .588 | | | .556 | .093 | .911 | .026 | .974 | | 2.703 | .069 | | 2.571 | | .078 |
|  |  | R | .042 | .838 | | .107 | | | .744 | .005 | .943 | .043 | .835 | | 1.429 | .233 | | 1.697 | | .194 |
|  | rs4570625 | A | .726 | .484 | | 1.881 | | | .154 | 2.989 | **.052** | 1.290 | .277 | | 2.632 | .073 | | 1.565 | | .211 |
|  |  | D | 1,449 | .230 | | 3.325 | | | .069 | .699 | .404 | 2.557 | .111 | | 4.167 | **.042** | | 1.755 | | .186 |
|  | rs4290270 | A | .447 | .640 | | 1.553 | | | .213 | 2.124 | .121 | .120 | .887 | | .855 | .426 | | .341 | | .711 |
|  |  | R | .077 | .782 | | 2.864 | | | .092 | 1.225 | .269 | .231 | .631 | | .865 | .353 | | .560 | | .455 |
| *OXTR* | rs53576 | A | .448 | .639 | | .877 | | | .417 | .335 | .716 | .005 | .995 | | .594 | .553 | | 1.541 | | .216 |
|  |  | R | .036 | .849 | | 1.269 | | | .261 | .008 | .927 | .018 | .893 | | 1.037 | .309 | | 2.617 | | .107 |
|  | rs968389 | A | .868 | .421 | | .530 | | | .589 | .527 | .591 | .199 | .819 | | .255 | .775 | | 1.459 | | .234 |
|  |  | D | .272 | .603 | | .003 | | | .958 | .893 | .346 | .558 | .456 | | .098 | .755 | | .926 | | .337 |
|  | rs2268498 | A | .825 | .439 | | 1.848 | | | .159 | .558 | .573 | 1.801 | .167 | | .386 | .680 | | .835 | | .435 |
|  |  | D | .110 | .740 | | .003 | | | .956 | .585 | .445 | 1.683 | .195 | | .184 | .668 | | .604 | | .438 |
| *OPRM1* | rs1799971 | A | 2.042 | .131 | | .587 | | | .556 | 1.316 | .270 | .781 | .459 | | 2.010 | .136 | | .911 | | .403 |
|  |  | D | .820 | .366 | | .745 | | | .389 | 2.047 | .153 | .384 | .536 | | .367 | .545 | | .385 | | .535 |
|  | rs677830 | A | .495 | .610 | | .397 | | | .673 | .725 | .485 | .490 | .613 | | .351 | .704 | | 2.721 | | .067 |
|  |  | D | .143 | .705 | | .556 | | | .457 | 1.443 | .231 | .057 | .811 | | .031 | .861 | | .921 | | .338 |
| *BDNF* | rs6265 | A | 1.948 | .144 | | 2.258 | | | .106 | 1.659 | .192 | 3.039 | **.049** | | 1.124 | .326 | | .506 | | .604 |
|  |  | D | .159 | .690 | | 3.429 | | | .065 | 2.111 | .147 | 3.842 | **.051** | | 1.910 | .168 | | .555 | | .457 |
|  | rs28722151 | A | .116 | .891 | | .620 | | | .538 | .532 | .588 | 1.764 | .173 | | 1.725 | .180 | | .330 | | .719 |
|  |  | R | .096 | .757 | | .983 | | | .322 | .002 | .963 | 3.434 | .065 | | 1.092 | .297 | | .015 | | .901 |
|  | rs11030101 | A | .207 | .813 | | .388 | | | .679 | .388 | .676 | 3.362 | **.036** | | 1.074 | .343 | | .167 | | .846 |
|  |  | R | .017 | .896 | | .751 | | | .387 | .676 | .411 | 6.495 | **.011** | | .855 | .356 | | .175 | | .356 |

*Notes:* Significant (*p* < .0036) and nominally significant results (*p* = .0036 - .05) are presented in bold. A-Additive model, R-Recessive model, D-Dominant model*.* Additive model was not calculated for *OPRM1* rs1799971 due to rare reference genotype. SEEK – SEEKING, SAD - SADNESS.

**Figure S1. Heatmap displaying associations between the primary emotions and the included polymorphisms on the total sample while controlling for age (-log10(p))**


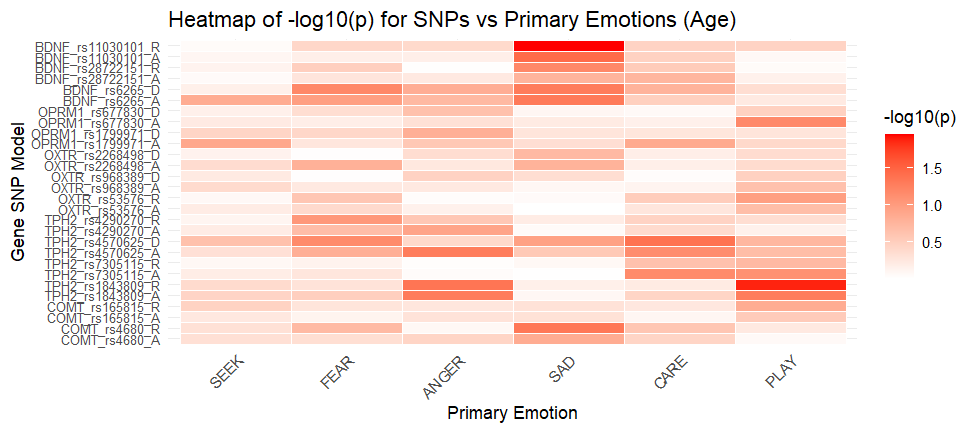

Supplement: Supplementary file 1 [file Supplementaryfile1.docx]
